# Energy intake and physical activity over the course of pregnancy and gestational weight gain: a systematic review and dose-response meta-analysis of data from randomized controlled lifestyle intervention trials

**DOI:** 10.1186/s12937-025-01182-w

**Published:** 2025-08-07

**Authors:** Yixin Chen, Sasithorn Sarnthiyakul, Sophie K. F. Michel, Chuyue Wu, Hans Hauner, Ondine S. von Ehrenstein, Jihong Liu, Liwei Chen

**Affiliations:** 1 Department of Epidemiology, Fielding School of Public Health, University of California, Los Angeles, Los Angeles, CA USA; 2 Institute of Nutritional Medicine, School of Medicine and Health, Technical University of Munich, Munich, Germany; 3 Department of Community Health Sciences, Fielding School of Public Health, University of California, Los Angeles, Los Angeles, CA USA; 4 Epidemiology and Biostatistics, Arnold School of Public Health, University of South Carolina, Columbia, SC USA

**Keywords:** Gestational weight gain, Energy intake, Systematic review, Dose-response meta-analysis

## Abstract

**Background:**

This systematic review and meta-analysis aimed to quantify the relationship between energy intake (EI) and physical activity (PA) during pregnancy and gestational weight gain (GWG) in randomized controlled trials (RCTs).

**Methods:**

RCTs measuring EI and PA at least twice and total GWG during pregnancy were eligible. To synthesize PA measures reported on different scales, standardized mean change per day (SMC/day) were obtained by dividing the change in PA by the standard deviation of the change. We estimated mean changes in EI, PA, and mean total GWG across studies, accounting for clustering within studies. One-stage dose-response meta-analyses (DRMA) quantified the additional GWG associated with changes in EI and PA during pregnancy.

**Results:**

A total of 21 RCTs with 7,705 participants were included. The mean total GWG was 11.99 kg (95% CI: 11.05 kg to 12.94 kg). The mean baseline EI was 1977 kcal/day across studies (range: 1652 to 2777 kcal/day) and the mean increase in EI throughout pregnancy was 132 kcal/day (95% CI: 54 to 209 kcal/day). The average change in PA during pregnancy was − 0.11 SMC/day (95% CI: -0.33 to 0.12 SMC/day). DRMA indicated 0.30 kg additional weight gain per 100 kcal/day increase in EI (95% CI: -0.01 kg to 0.60 kg, *P* = 0.06). The effect size was greater in studies with low risk of bias vs. high risk of bias (0.57 vs. -0.20 kg, P for difference = 0.02). DRMA showed 0.24 kg less weight gain per 0.25 SMC/day increase in PA (-0.50 to 0.02 kg, *P* = 0.07).

**Conclusions:**

Average GWG often exceeds recommendations of current guidelines, particularly among women with overweight/obesity (OWOB), while average increases in EI were below current recommendations, and PA levels were frequently observed to decrease. DRMA further suggests that GWG may be modifiable through changes in EI and PA with greater EI increases or PA reductions linked to greater GWG, especially among women with OW/OB. Despite challenges in precisely quantifying these associations, integrated findings from this comprehensive systematic review and subgroup/sensitivity analyses highlight the need for more individualized nutrition and exercise recommendations and may warrant revisiting current guidelines.

**Supplementary Information:**

The online version contains supplementary material available at 10.1186/s12937-025-01182-w.

## Background

It is well-documented that excessive gestational weight gain (GWG) is associated with multiple adverse outcomes in women and their offspring [1, 2]. Specifically, excessive GWG has been established as a risk factor for preeclampsia, gestational diabetes, and postpartum weight retention, thereby directly contributing to a higher risk of obesity, type 2 diabetes mellitus, and cardiovascular diseases in women later in life [3–11]. Also, in the offspring, excessive GWG is associated with higher risks of preterm births, macrosomia, large for gestational age (LGA) as well as childhood or adult obesity and insulin resistance [12–21]. To achieve optimal health, the Institute of Medicine (IOM; currently known as The National Academy of Medicine or NAM) developed GWG guidelines based on maternal pre-pregnancy body mass index (BMI) in 2009 [22]. Current recommendations suggest weight gain of 13–18 kg for underweight BMI (< 18.5), 11–16 kg for normal weight BMI (18.5–24.9), 7–11 kg for overweight BMI (25.0–29.9) and 7–9 kg for obese BMI (≥ 30.0) [22]. Unfortunately, the prevalence of excessive GWG is still rather high, with 48–50% in the United States (U.S.) [23, 24], and 27.8% globally [25].

The dietary total energy intake (EI) during pregnancy is a critical factor for GWG, however, the ideal levels of EI in achieving appropriate GWG are an issue of ongoing debate. In 1985, the World Health Organization (WHO) released a report that the estimated total additional energy required during pregnancy is 80,000 kcal (335,000 kJ) [26]. This estimation is based on factorial calculations of a theoretical model developed by Hytten and Chamberlain in the 1970s and 1980s, assuming an average GWG of 12.5 kg [27, 28]. Since then, revision of the calculations has been made based on several longitudinal experimental studies on energy balance in pregnancy. With these experimental human data, Butte and King in 2005 estimated that the total extra energy requirement of pregnancy was 77,680 kcal (325,000 kJ) for women with a mean GWG of 12.0 kg, distributed as 89 kcal (375 kJ), 287 kcal (1200 kJ), 466 kcal (1950 kJ) per day in the 1st, 2nd, and 3rd trimesters, respectively [29]. However, current guidelines vary between sources and countries, for example, the American College of Obstetricians and Gynecology (ACOG), as well as dietary guidelines published by the US Department of Agriculture (USDA) recommend 0 kcal, 340 kcal and about 450 kcal per day increases per trimester [30, 31], while in the UK, an increase by only 200 kcal/day, and only in the third trimester, is recommended [32].

These recommendations for EI during pregnancy may be problematic and need an update for several reasons. First, it is well acknowledged that EI for pregnant women should be population-specific, considering critical factors such as pre-pregnancy body size. However, the theoretical models did not consider pre-pregnancy body size in their calculation [27–29]. For women with pre-pregnancy overweight or obesity (OWOB), a 12 kg GWG would be excessive, thus further increases in EI may not be necessary [33]. Second, the theoretical models were developed based on studies between the 1980s and 1990s [29]. PA levels, a dominant and most variable component of energy cost, have decreased over time, both in the U.S [34–36]. and globally [37]. While the WHO and Centers for Disease Control (CDC) recommends that pregnant women perform at least 150 min of moderate-intensity PA throughout the week, without a need to reduce previous levels of PA [38], a recent U.S. study with a nationally representative sample found that only 73% pregnant women reported meeting the recommended PA levels [39]. Also, multiple observational studies found that women reduced their PA by about 120 MET min/week from early to late pregnancy [40, 41]. A global study by Guthold et al., 2018 also illustrates an increase in prevalence of inactivity specifically for women in high-income countries from 2001 to 2016 [42]. Third, pregnant women nowadays are on average older in many countries, which would also affect ideal levels of EI for pregnant women [43].

Indeed, a meta-analysis of observational studies indicated that the current generation of pregnant women in developed countries actually increase their EI by only about 100–200 kcal per day, but excessive GWG is still highly prevalent in these countries [33]. Other systematic reviews and meta-analyses based on observational studies came to similar conclusions [44]. Taken together, available evidence tends to suggest that the recommended EI may not be appropriate for current pregnant populations.

Therefore, we aimed to quantify the relationship between changes in EI and PA during pregnancy and GWG, using dose-response meta-analysis (DRMA), while stratifying by pre-pregnancy BMI and other important covariates.

## Methods

This review was performed according to a pre-specified protocol (CRD42023243289) and adhered to the updated Preferred Reporting Items for Systematic Reviews and Meta-Analyses (PRISMA) guidelines of 2020 [45].

### Literature search strategy

We developed our systematic literature search strategies based on a preliminary scoping search yielding 29 primary studies and 4 systematic reviews (Supplemental Material S1: References identified during preliminary scoping). We identified both keywords and MeSH terms commonly used in these references and tabulated them using the PubMed Reminer tool [46]. We also consulted MeSH entry terms to identify additional synonyms and used the SearchRefiner tool from the Systematic Review Accelerator website to assess and increase the sensitivity and specificity of our search strategy against the 29 identified primary studies [47]. We applied the above search strategies to 6 electronic databases, including PubMed, Embase, Web of Science, PubAg, CINAHL, and the Cochrane Central Register of Controlled Trials (CENTRAL) on publications until April 23th 2021 [48]. The databases were searched without language, publication status, or publication date restrictions. We combined all search strategies using controlled vocabulary terms and text words through Boolean operators (Supplemental Material S2: Full electronic search strategies). Additionally, we performed internet searches using Google and Google Scholar as well as backward and forward citation searches. References that appeared eligible based on the title were cross-referenced against the library of references obtained from the electronic databases.

### Study selection

To be included in this systematic review, a study had to be a randomized controlled trial (RCT) in design and reported EI, PA, and GWG as continuous variables at two time points (pre- or early-pregnancy and one follow-up later in pregnancy) (see Supplemental Material S3, inclusion criteria). Further, we only considered studies that also reported pre-pregnancy BMI. We excluded studies if they focused only on (1) non-adults (i.e., < 18 years), (2) women with pre-pregnancy BMI < 18.5 (i.e., underweight), (3) women carrying multiple fetuses, or (4) women with pre-pregnancy diabetes (i.e., likely to be advised a special diet). We excluded studies focused solely on women with underweight, as important population (e.g., overall health, metabolic condition) and contextual (e.g., systemic food insecurity) differences make such studies incompatible with the lifestyle intervention studies we sought. For example, women with underweight may have significantly different energy requirements, are recommended higher GWG, and may exhibit different physiological responses [49, 50]. Three reviewers (SC, SS, CW), evaluated all studies in duplicate by applying the inclusion and exclusion criteria, starting with a title and abstract screening, followed by a full-text screening. When there were disagreements in reviewers’ decisions regarding eligibility, references were included into the subsequent screening stage. Conflicts unresolved after full text screening were resolved through discussion with the review team. Identified references were collated, de-deduplicated, and screened using EndNote software X8 (Thomas Reuters, New York City, NY).

### Data extraction

We extracted data with an internally developed, pilot-tested protocol. Data extraction of each study was performed by at least two independent reviewers (CW, SC, SS). Information on study design, study year and country, sample size, study population, content of lifestyle intervention, gestational age at baseline and final study visit, definition of GWG, method and timing of assessment of EI, PA, and GWG, and aggregated data on pre-pregnancy BMI, EI, PA, and GWG were extracted. We contacted study authors if publications indicated that relevant data on EI, PA, or GWG were measured, but not reported, or if missing or unclear information was identified (*n* = 7) [51–57]. If data was only reported in figures (*n* = 1) [57], and could not be obtained after making requests to study authors, it was extracted using the WebPlotDigitizer tool [58].

### Risk of bias assessment

Studies were assessed based on criteria combining elements of the revised Cochrane risk-of-bias tool for individual or cluster-RCTs [59] and the ROBINS-E tool [60] (Supplemental Material S4: Table of Cochrane’s ROB 2 tool domains modified using ROBINS-E tool components). Therefore, our assessment included potential risk of bias arising from issues with (1) the randomization process, (2) the timing of recruitment of participants (only for cluster-RCTs), (3) the measurement of the exposures (EI and EE at two timepoints), (4) the measurement of the outcome (total GWG), (5) level of missing data, and (6) the selection of the reported results. For the measurement of EI, use of food frequency questionnaires (FFQ) was graded as “some concern” because FFQ only assess dietary intake qualitatively or semi-quantitatively, whereas 24-hour dietary recalls and food records were rated as a “low” risk of bias [61]. For PA, objective measurements such as accelerometers were rated as a “low” risk of bias, whereas subjective methods such as PA questionnaires were rated as “some concern”. For the measurement of the GWG, only studies using a measured final weight (i.e., weight in delivery room or weight measured at last clinic visit) to compute total GWG were considered at a “low” risk of bias. If a study had over 20% missing data on exposure or outcome, it was considered to be at a “high” risk of bias. If the study had over 10% but less than 20% missing data, it was considered to be of “some concern.” Summary figures were prepared using the *robvis* tool [45].

### Statistical analysis

R Studio software version 2022.07.1 (RStudio Inc., Boston, MA, USA) was used for all analyses. P-values < 0.05 were considered statistically significant. For tests of subgroup differences, p-values < 0.1 were considered statistically significant.

Raw mean change (RMC) in EI and standardized mean change (SMC) [62, 66] in PA during pregnancy and corresponding variances were estimated using the raw mean values and variances in each study group at baseline (T1) and latest assessed time points (T2), and combined with correlation coefficients applied to these repeated measurements (0.60 and 0.48 were used for EI, and 0.75 and 0.76 were used for PA) based on studies for which we had access to the required data [56, 65] and previous meta-analyses [44]. For EI, we were able to conduct analyses using the raw measure of changes in caloric intake per day (kcal/day). However, for PA, we needed to use the standardized mean change (SMC), because the included studies used various measures for total/ leisure PA (e.g., steps per day, PA score, MET hours/ week, etc.). Therefore, prior to meta-analysis, standardized mean changes (SMC) per day were calculated by dividing the change in PA within each study group by the standard deviation of the change [62, 63]. The standard deviation of the change was estimated, based on standard deviations at baseline and follow-up, as well as imputed within-group correlations [64], based on included studies reporting the required data. Further details are given in Supplemental Material S5: Statistical analyses [67, 68]. To increase interpretability, transforming back to a familiar scale was done by multiplying the SMC in PA by the pooled standard deviations of change in studies using the desired original scale and among the largest/ most representative of the primary studies [69, 70].

For descriptive analyses, means and variances in (a) baseline EI, (b) RMC in EI and SMC in PA over the course of pregnancy and (c) total GWG of each study group were pooled, while accounting for the clustering of study groups within studies via three-level, random effects meta-analytical models, using “*metamean*” in R. In addition, we also ran the above analyses separately for the intervention vs. control groups. Heterogeneity between studies was assessed based on I^2^ statistics and interpreted according to the Handbook of the Cochrane Collaboration [71].

We then estimated the mean difference in GWG (kg) by the differences in changes in EI (kcal/day) and PA (SMC/day) during pregnancy across studies using a one-stage, random-effects DRMA model, using the “*dosresmeta”* package [72–74]. The exposure/dose (i.e., mean change in EI, SMC in PA) values in the design matrix are constructed so that the reference values are always re-centered to be zero. This is particularly relevant when the reference values vary across studies, and/or for non-zero reference doses [75], which both applied to our analyses. Similarly, the outcome GWG was re-centered and expressed as a contrast (difference in means) between the index and reference groups. Thus, the model has no intercept and random slopes [73, 75]. We also assessed both linear and quadratic models.

*Post hoc* considerations included graphical inspection of the data and the goodness-of-fit measures of the various models [73, 76]. Statistical heterogeneity between primary studies was assessed using a variance partition coefficient (VPC) [77], as this allows for the examination of heterogeneity across the exposure range, which makes it a more useful measure for DRMA, compared to singular statistics such as I^2^ [73].

### Subgroup analyses and sensitivity analyses

For both the meta-analyses of mean changes in EI, SMC in PA, and mean GWG, as well as for the DRMA, we performed subgroup analyses based on the following study-level variables, as appropriate: (1) pre-pregnancy BMI inclusion criteria (i.e., studies including only women with OWOB vs. studies including all women); and (2) country group (i.e., countries in the West vs. East). Turkey was classified as Western, as the country has undergone significant Westernization of its diet, with increased consumption of processed foods, fast food, and sedentary behavior, trends more aligned with Western lifestyles [78]. In contrast, Iran, while also experiencing nutrition transition, retains more traditional dietary patterns, such as higher reliance on home-cooked meals, lower fast food consumption, and greater emphasis on family-based eating [79]. Also, Iranian studies report gender-based constraints on PA, leading to higher rates of sedentary behaviors among women [80], suggesting that lifestyle factors remain influenced by political and sociocultural norms, and thus Iran was classified as Eastern. We also assessed the role of (3) EI and PA assessment methods (i.e., “high/some concern” vs. “low” risk of bias), and (4) missing data (i.e., more than 10%, and more than 20% of either exposure or outcome data missing), and (5) whether studies used self-reported pre-pregnancy weight or measured weight in first or second trimester to estimate total GWG. Tests of subgroup differences were performed for the DRMA.

For the descriptive analyses and the DRMA, we conducted several sensitivity analyses as appropriate, including (1) excluding each study from the meta-analyses (“leave-one-out analyses”), (2) excluding the studies which assessed EI, PA, or GWG only to mid-pregnancy; (3) applying the different correlation coefficients for EI and PA, ranging from 0.35 to 0.76, and (4) using sample sizes adjusted for cluster-level randomization in certain studies, and excluding cluster-RCTs altogether.

## Results

**Fig. 1:**
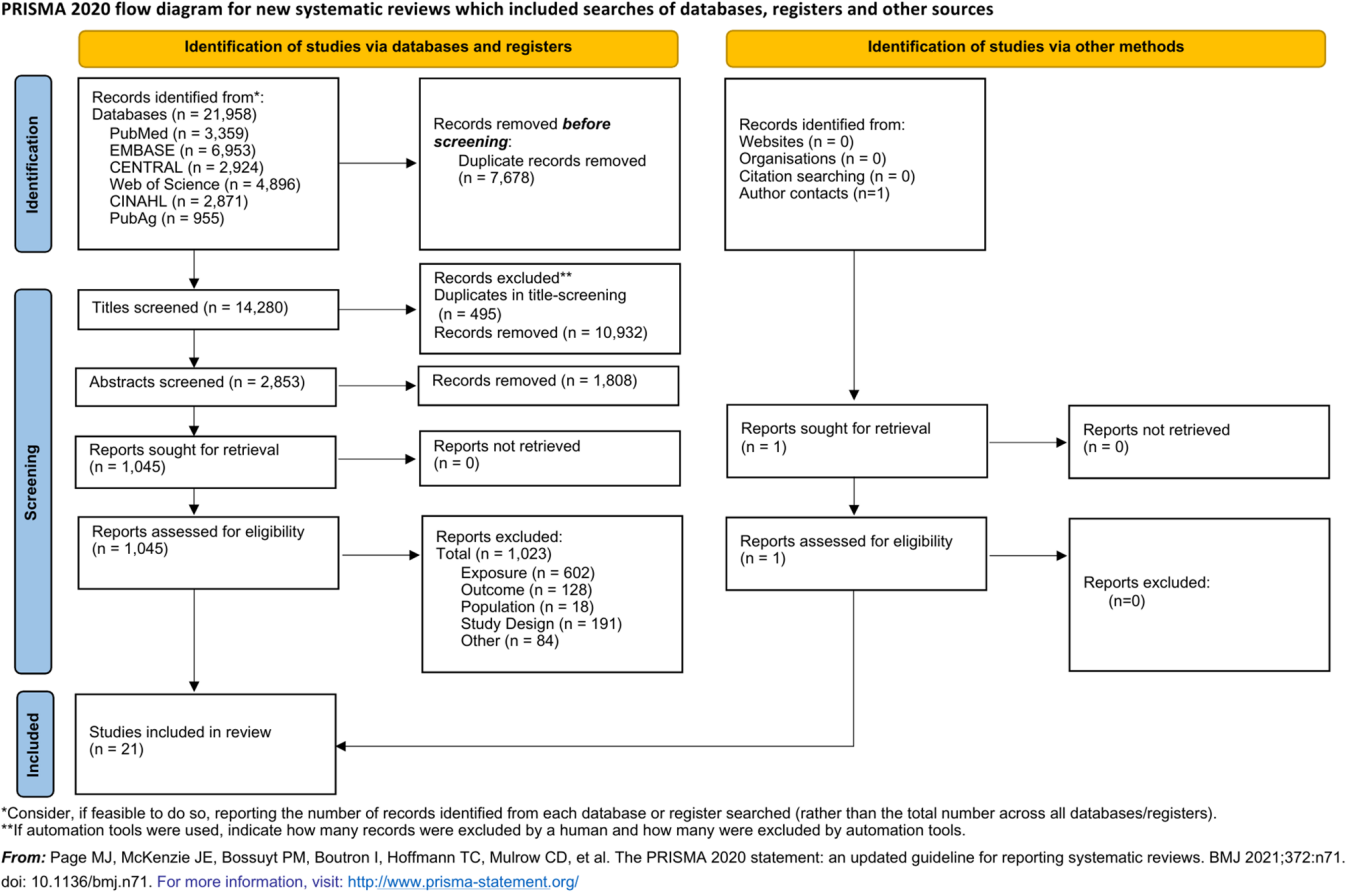
PRISMA flow diagram for the selection of primary studies

We identified 14,280 reference titles during the initial search and found 1,045 full-text articles that met the inclusion and exclusion criteria for this systematic review. We identified 21 RCTs with relevant data reported in 24 journal articles (Fig. 1) [56, 57, 65, 81–101]. Additional data was retrieved from study authors for one study, where we were able to obtain EI and PA measurements at 32 week’s gestation and measured weight at delivery [56]. 

**Table 1:** Characteristics of included studies

Study, Author, Publication Year	Country	*N* (Intervention/control)	Study population BMI category	Baseline/final measurement of EI/PA (GW)	Intervention targets/format	EI assessment method	PA assessment method	Definition of GWG
Guelinckx et al., 2010 [89]	Belgium	65/65	OB	< 15/>28	Diet, PA, and GWG monitoring (advice)	7-day dietary records	Questionnaire (Baecke) [102]	The difference between weight at delivery and self-reported pre-pregnancy weight
NELLI trial Luoto et al., 2011 [96]	Finland	219/180	OW/OB	Pre-pregnancy 36–37	Diet and PA (individual counseling at 5 antenatal visits)	FFQ	Questionnaire (unknown)	The difference between weight during 36–37 GW and self-reported pre-pregnancy weight
Hui et al., 2012 [94]	Canada	112/112	All	< 26/2 months later	Diet and PA	3-day dietary records	Questionnaire (PARMed-X form)	The difference between weight in the delivery room and pre-pregnancy weight
FeLIPO trial Rauh, K., et al., 2013 [100]	Germany	167/83	All except UW	16–18/36–38	Diet, PA, and GWG monitoring (structured counseling sessions)	7-day dietary records	Questionnaire (IPAQ long version)	The difference between weight during 36–38 GW and self-reported pre-pregnancy weight
LIMIT trial Dodd, J. M., et al., 2014 [85]	Australia	1108/1104	OW/OB	10–20/36	Diet and PA (behavioral intervention)	FFQ	Questionnaire (SQUASH)	The difference between weight during 36 and 10–20 GW.
Hui, A. L., et al., 2014 [57]	Canada	57/56	All	< 20/2 months later	Diet and PA	3-day dietary records	Questionnaire (PARMed-X form)	The difference between weight at delivery and self-reported pre-pregnancy weight
Jing et al., 2015 [95]	China	115/106	All	~ 12/20–24	Personalized interventions based on the health belief model	FFQ	Questionnaire (PPAQ)	The difference between last measured weight during 20–24 GW and baseline (12 GW)
UPBEAT trial Poston et al., 2015 [99]	UK	783/771	OB	15–18/27–28	Diet and PA (behavioral intervention)	FFQ	Questionnaire (IPAQ)	Final measurement timepoint not reported, pre-pregnancy weight used for baseline weight
Asci et al., 2016 [81]	Turkey	51/51	All	12–15/37	Diet, PA, and GWG monitoring	3-day dietary records	Questionnaire (Profile-II PA subscales)	The difference between measured weight at 37 GW and measured weight at 12–15 GW.
Smith et al., 2016 [101]	US	26/25	All except UW	10–14/34–36	PA (behavioral intervention and social support	3-day dietary records	Accelerometer (SenseWear Mini armband for 1 week)	The difference between last measured weight at 34–36 GW and self- reported pre-pregnancy weight
Chan et al., 2018 [83]	China (Hong Kong)	118/111	OW/OB	$\:\le\:$12/24–28	Diet and PA (Dietitian-led)	3-day dietary records	Questionnaire (Chinese version IPAQ)	The difference between measured weight at delivery and the self-reported pre-pregnancy weight
Phelan et al., 2018 [98]	US	132/132	OW/OB	9–16/35–36	Diet and PA (behavioral intervention with partial meal replacement)	24-hour dietary recalls (2 days)	Accelerometer (ActiGraph GT3X) (1 day)	The difference between measured weight at 35–36 GW and self- reported pre-pregnancy weight
Buckingham-Schutt et al., 2019 [82]	US	27/29	All except UW	8–14/34–36	Diet and PA (behavioral intervention)	3-day dietary records	Accelerometer (Wearable fitness tracker armband for 1 week)	The difference between measured weight at 34–36 GW and self-reported pre-pregnancy weight
OPTIMISE trial, Dodd et al., 2019 [86]	South Australia	323/322	NW	10–20/28–36	Diet (advice)	FFQ	Questionnaire (SQUASH)	The difference between measured weight at 36 GW and measured weight at 10–20 GW
GeliS trial, Günther et al., 2019 [90]	Germany	1061/1040	All	12–16/ after 29	Diet, PA, and GWG monitoring (behavioral intervention)	FFQ	Questionnaire (PPAQ)	The difference between measured weight at last prenatal visit and the measured weight at the first prenatal visit
Haijian et al., 2020 [91]	Iran	33/33	OW	16–20/35–37	Diet, PA, and GWG monitoring (Counseling)	FFQ	Questionnaire (IPAQ)	The difference between weight at delivery from medical files and baseline weight from medical files
PLAN trial, Huang et al., 2020 [93]	Australia	30/27	All	6–11/18–23	Diet, PA and well-being (advice through website)	3-day food records	Accelerometer (ActiGraph Bluetooth Smart wGT3X for 1 week)	The difference between the last recorded weight in pregnancy (36 GW) and the first recorded weight (6–11 GW)
GLOW trial, Ferrara et al., 2020 [65]	US	200/198	OWOB	10–32	Diet, PA, and stress management (behavioral intervention through telehealth)	24-hour dietary recalls (3 days)	Accelerometer (ActiGraph wGT3X-BT, 7 days)	The difference between last measured pregnancy weight (38.4 GW) and measured pre-pregnancy weight, or weight at 10 GW
Ding B et al., 2021 [84]	China	104/111	OW/OB	< 12/24–28	Diet and PA (advice delivered through WeChat app)	24-hour dietary recall (1 day)	Pedometer (Smartphone, 7 days)	The difference between weight at delivery and self-reported pre-pregnancy weight
Healthy Mom Zone trial Downs et al., 2021 [88]	US	15/16	OW/OB	10/ ~ 36	Adaptive intervention with Diet, PA, and GWG minoring (face-to-face counselling weekly plus behavioral intervention)	Back-calculation equation of EI	Accelerometer (Jawbone UP3) (daily)	The difference between weight at ca. 36 GW and 8–12 GW measured using a Wi-Fi scale
HIPP trial, Liu et al., 2021 [56]	US	114/114	OW/OB	< 16/32	Diet, PA, and GWG monitoring (behavioral intervention)	24-hour dietary recalls (2 days)	Accelerometer (SenseWear armband)	The difference between medically abstracted weight at delivery and self-reported pre-pregnancy weight. If weight at delivery was unavailable, last measured weight during pregnancy was used (38.5 GW)

Note: All participants in the control group were provided with usual prenatal care

BMI categories: normal weight (NW, BMI: 18.5–24.9 kg/m^2^); Overweight (OW: BMI: 25.0–29.9 kg/m^2^); Obese (OB: BMI: ≥30.0 kg/m^2^)

Abbreviations: BMI: Body mass index, EI: energy intake, FFQ: food frequency questionnaire, GDM: Gestational diabetes mellitus, GW: weeks of gestation, GWG: Gestational weight gain, IPAQ: International Physical Activity Questionnaire, NA: Not reported and not available, NW, Normal weight, OB: Obesity, OW: Overweight, PA: Physical activity, PPAQ: The Pregnancy Physical Activity Questionnaire, SQUASH: Short Questionnaire to Assess Health-enhancing Physical Activity, UK: The United Kingdom, US: The United States, UW: Underweight

### Characteristics of the studies

The characteristics of the included studies are summarized in Table 1. Of the 21 RCTs, 17 performed randomization at the individual participant level [56, 57, 65, 81–86, 88, 89, 93–95, 97, 99, 101], while 4 being randomized at obstetric or healthcare facility level (i.e., cluster-RCTs) [90, 91, 96, 100]. The sample size ranged from 31 to 2212 for individual RCTs [56, 57, 65, 81–86, 88, 89, 93–95, 97, 99, 101], while it varied from 66 to 2102 for cluster RCTs [90, 91, 96, 100]. These studies were conducted across different countries, with 8 taking place in North America [56, 57, 65, 82, 88, 94, 97, 101], 5 in Europe [89, 90, 96, 99, 100], 3 in Australia [85, 86, 93], 2 in the Middle East [81, 91], and 3 in China [83, 84, 95]. Overall, 17 studies were from the “West” [56, 57, 65, 81, 82, 85, 86, 88–90, 93, 94, 96, 97, 99–101], while the rest 4 were considered from the “East” [83, 84, 91, 95]. Four studies did not provide racial and ethnic information [83, 91, 96, 100], while studies conducted in North America, especially in the United States (US), included participants from diverse racial and ethnic groups, whereas studies conducted in Australia and Europe predominantly reported participants of Caucasian descent.

Studies primarily targeted and enrolled pregnant women aged 18 years or older, although some also included younger women, at < 20th gestational weeks (GW), except for one study, which included participants before the 26th GW [94]. Among the selected studies, 4 excluded women who were underweight before pregnancy [82, 90, 100, 101], 1 study included women with normal weight (NW) exclusively [86], 7 included women with (OWOB) [56, 65, 83, 85, 88, 96, 97], 1 included only women with overweight [91], and 2 studies included only women with obesity [89, 99]. Further, 2 studies had eligibility criteria for baseline BMI of either ≥ 20 kg/m^2^ [93], or 24 kg/m^2^ [84], respectively. Four of the RCTs did not have specific eligibility criteria concerning weight or BMI [57, 81, 94, 95].

Dietary intake assessment methods varied across studies. Nine studies used food records [57, 81–83, 89, 93, 94, 100, 101], 4 used 24-hour dietary recalls [56, 65, 84, 97], 7 used FFQs to assess dietary intake [85, 86, 90, 91, 95, 96, 99], and 1 study used the participant’s weight and PA to back-calculate EI [88]. For the assessment of PA, 8 used objective methods such as pedometers [56, 65, 82, 84, 88, 93, 97, 101], while 13 studies used PA questionnaires [57, 81, 83, 85, 86, 89–91, 94–96, 99, 100]. GWG was defined as the difference of either self-reported pre-pregnancy weight, or first measured weight (e.g., recorded at first prenatal appointment or at trial entry), and the last measured weight (e.g., recorded at the last obstetric visit or at delivery).

### Qualitative assessment of bias

**Fig. 2:**
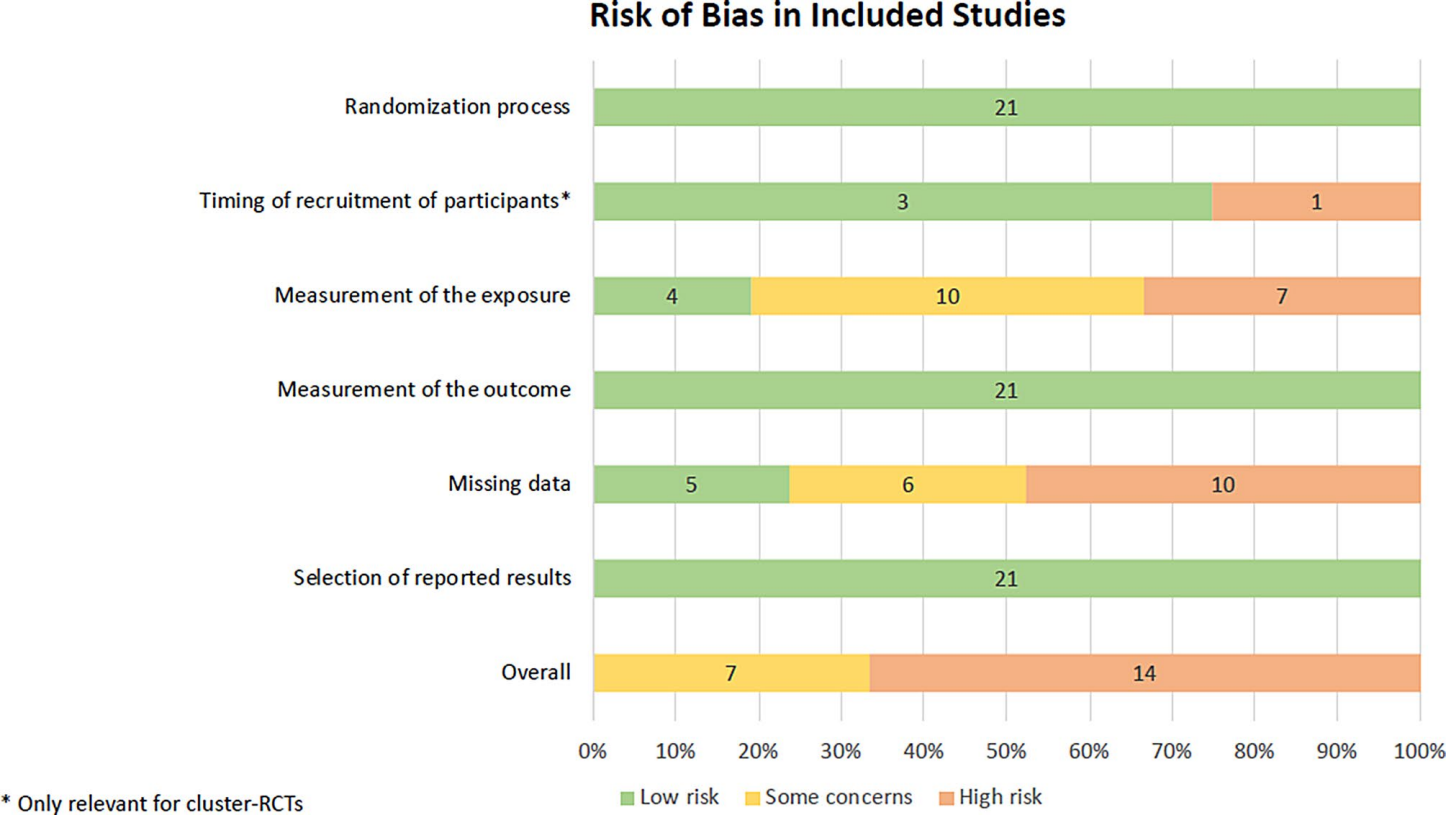
Risk of bias in included RCTs. Abbreviations: RCT: randomized controlled trial

Figure 2 summarizes our assessment of the risk of bias in all 21 studies. A more detailed overview including the ratings for each domain for each study was included in the supplemental materials (Supplemental Material S6: Individual risk of bias assessment results). Seven studies were judged to be of “some concern”, and 14 studies were considered at a “high” risk of bias. Concerns were largely related to the valid and accurate measurement of EI or PA, as well as missing data, where up to 53% of data were missing [94].

### Descriptive meta-analyses of EI and PA during pregnancy and total GWG

**Fig. 3:**
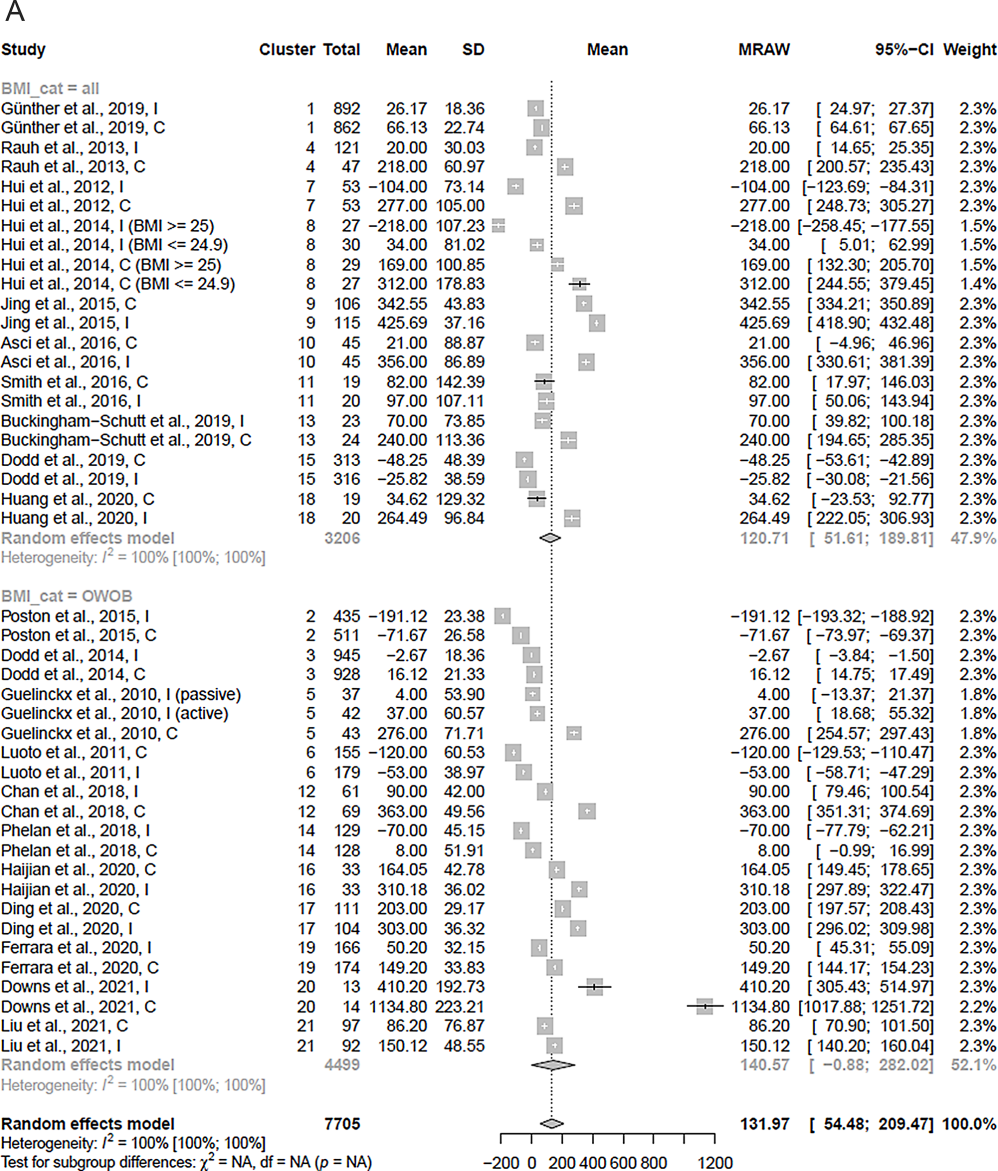

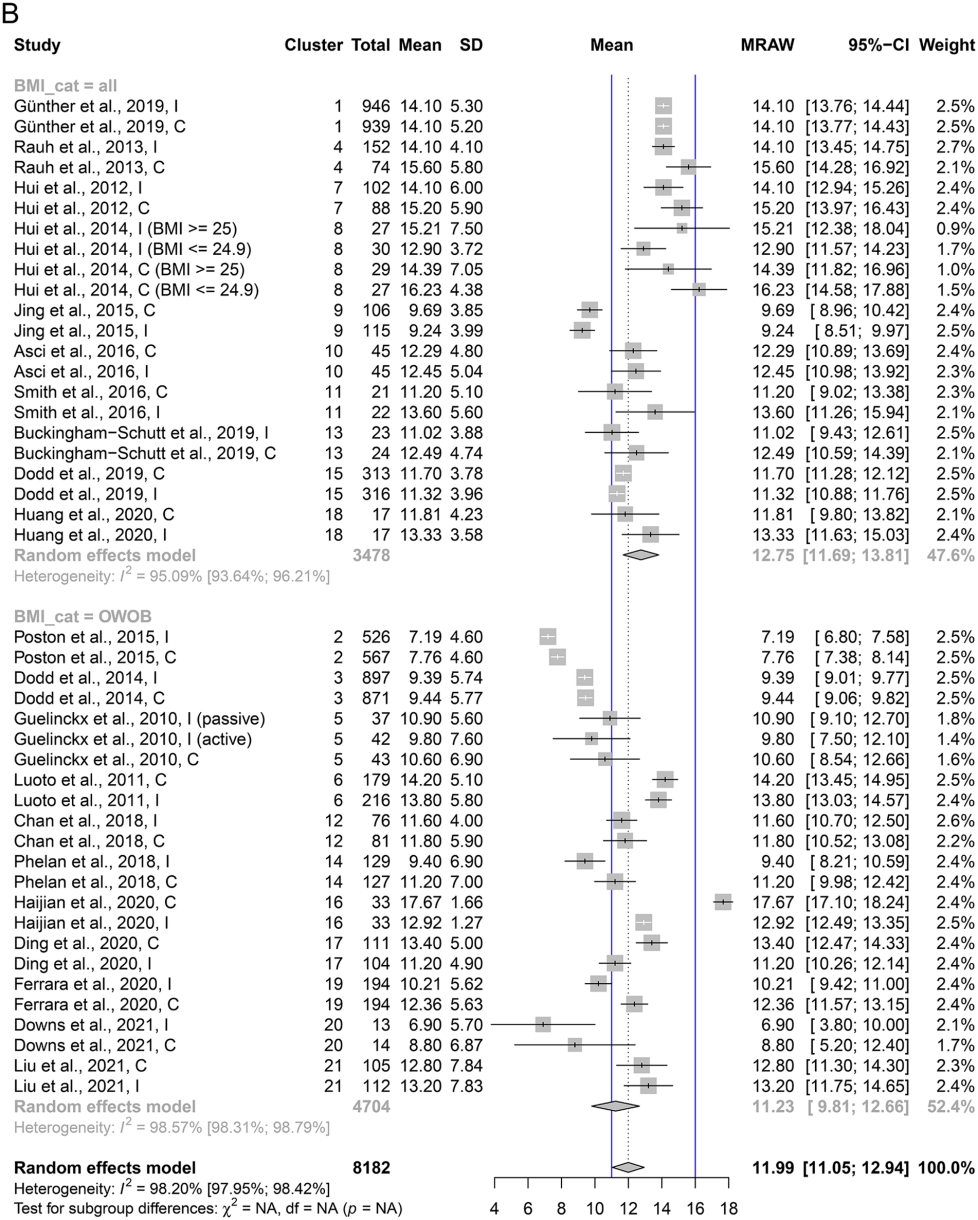
**A**. Pooled change in EI (raw mean change) across studies (kcal/day). The analysis accounted for clustering of study groups within studies (2–4 groups per study) via three-level meta-analysis, stratified by BMI group inclusion criteria. Abbreviations: SD: standard deviation, MRAW: raw mean, CI: confidence interval, BMI: body mass index, OWOB: overweight and obese **B**. Pooled mean GWG (kg) across studie. The analysis accounted for clustering of study groups within studies (2–4 groups per study) via three-level meta-analysis, stratified by BMI group inclusion criteria. The blue lines indicate the NAM’s upper threshold of recommended GWG for women with normal weight (16 kg) and women with overweight (11 kg). Women with obesity are recommended to gain no more than 7 kg. Abbreviations: SD: standard deviation, MRAW: raw mean, CI: confidence interval, BMI: body mass index, OWOB: overweight and obese

The baseline EI was 1977 kcal/day across all groups, 1967 kcal/day (95% CI: 1878 to 2056, I^2^ = 94%) among women randomized to lifestyle interventions, and 1980 kcal/ day (95% CI: 1890 to 2070, I^2^ = 94%) among control groups. Baseline EI was 1997 kcal/day (95% CI: 1907 to 2086 kcal/day) among studies including all BMI categories, vs. 1967 kcal/day (1814 to 2121 kcal/day) among studies including only women with OWOB. The pooled increase in EI was 132 kcal/day (95% CI: 54 to 209 kcal/ day, I^2^ = 100%) during pregnancy across all groups (Fig. 3A), 92 kcal/day across intervention groups (95% CI: 17 to 168 kcal/ day, I^2^ = 100%), and 173 kcal/day among control groups (95% CI: 66 to 280 kcal/ day, I^2^ = 100%) (Table 2). The pooled increase in EI was 121 kcal/day (95% CI: 52 to 190 kcal/ day, I^2^ = 100%) in studies including women in all BMI categories, 141 kcal/day (95% CI: -1 to 282 kcal/ day, I^2^ = 100%) in studies conducted in women with pre-pregnancy OWOB, 99 kcal/ day (95% CI: 12 to 186 kcal/day) in studies conducted in the West, and 275 kcal/ day (95% CI 197 to 353 kcal/day) in studies conducted in the East (Table 2). Excluding two studies that only reported changes in EI to mid-pregnancy (≤ 24 GW) [93, 95], changes in EI were estimated as 118 kcal/day (95% CI: 37 to 199 kcal/ day) (Supplemental Material S7: Sensitivity analyses of descriptive results). Among studies with less than 10% data, the estimate was 96 kcal/ day, 95% CI: -16 to 209 kcal, I^2^ = 100%, and among studies with less than 20% missing data, it was 171 kcal, 95% CI: 57 to 285 kcal, I^2^ = 100%. In sensitivity analyses testing different correlation values, excluding cluster RCTs, and evaluating the role of risk of bias in assessing caloric intake, the estimated change in EI was largely unchanged from the main analyses (Supplemental Material S7: Sensitivity analyses of descriptive results).

**Table 2:** Three-level meta-analysis and subgroup analyses of mean changes in EI and GWG over the course of pregnancy

	Main analysis (*N* study = 21)	Lifestyle intervention groups (*N* study = 21)	Control groups (*N* study = 21)	All BMI categories ^a^ (*N* study = 10)	Overweight/ obese BMI categories (*N* study = 11)	West ^b^ (*N* study = 17)	East (*N* study = 4)
Change in EI	132 kcal/ day 95% CI: 54 to 209 I^2^ = 100%	92 kcal/ day 95% CI: 17 to 168 I^2^ = 100%	173 kcal/ day 95% CI: 66 to 280 I^2^ = 100%	121 kcal/ day 95% CI: 52 to 190 I^2^ = 100%	141 kcal/ day 95% CI: -1 to 282 I^2^ = 100%	99 kcal/ day 95% CI: 12 to 186 I^2^ = 100%	275 kcal/ day 95%CI: 197 to 353 I^2^ = 100%
Change in PA	-0.11 SMC/ d 95% CI: -0.33 to 0.12 I^2^ = 100%	0.06 SMC/ d 95% CI: -0.21 to 0.33 I^2^ = 100%	-0.28 SMC/ d 95% CI: -0.49 to -0.06 I^2^ = 100%	-0.09 SMC/ d 95% CI: -0.45 to 0.25 I^2^ = 100%	-0.11 SMC/ d 95% CI: -0.42 to 0.20 I^2^ = 100%	-0.19 SMC/ d 95% CI: -0.41 to 0.04 I^2^ = 100%	0.24 SMC/ d 95% CI: -0.48 to 0.94 I^2^ = 100%
GWG	11.99 kg 95% CI: 11.05 to 12.94 I^2^ = 98%	11.60 kg 95% CI: 10.68 to 12.53 I^2^ = 98%	12.44 kg 95% CI: 11.37 to 13.51 I^2^ = 99%	12.75 kg 95% CI: 11.69 to 13.81 I^2^ = 95%	11.23 kg 95% CI: 9.81 to 12.66 I^2^ = 99%	11.93 kg 95% CI: 10.87 to 12.99 I^2^ = 98%	12.19 kg 95% CI: 9.83 to 14.56 I^2^ = 99%

^a^ BMI was based on the inclusion/exclusion criteria of each study. One study included only women with normal BMI and was grouped into the “All BMI” group

^b^ A study conducted in Istanbul in Turkey was classified as “Western” [81]

Abbreviations: BMI: body mass index, CI: confidence interval, EI: energy intake, GWG: gestational weight gain, PA: physical activity, SMC: standardized mean change

The pooled change in PA was − 0.11 SMC/day (95% CI: -0.33 to 0.12, I2 = 100%) across all groups, 0.06 SMC/day in intervention groups (95% CI: -0.21 to 0.33, I2 = 100%) and − 0.28 SMC/day among controls (95% CI: -0.49 to -0.06, I2 = 100%) (Table 2 and Supplemental Material S8, Forest plot). Changes in PA were nearly identical when stratifying studies based on BMI inclusion criteria. There was a 0.19 SMC/ day (95% CI: -0.41 to 0.04, I2 = 100%) decrease in PA levels observed in studies conducted in the West, and 0.24 SMC/ day increase (95% CI: -0.48 to 0.94, I2 = 100%) reported by studies conducted in the East. Testing different correlation coefficients for estimating SMC in PA, the estimate changed most notably to -0.06 SMC/ day (95% CI: -0.26 to 0.14, I2 = 100%) (Supplemental Material S7: Sensitivity analyses of descriptive results). Among studies with less than 10% missing data, the estimate was − 0.01 SMC, 95% CI: -0.55 to 0.53, I2 = 100%), and for 20% missing data, to 0.05 SMC/ day, 95% CI: -0.30 to 0.39, I2 = 100%). Studies with an objective PA measure reported a 0.02 SMC/ day decrease in PA (95% CI: 0.42 to 0.39, I2 = 100%), while studies using a subjective measure reported a 0.16 SMC/ day decrease (95% CI: -0.44 to 0.12, I2 = 100%). Excluding studies measuring PA only to mid-pregnancy did not significantly alter these results (Supplemental Material S7: Sensitivity analyses of descriptive results).

Overall, mean GWG ranged from 6.90 to 17.67 kg, with a pooled mean of 11.99 kg (95% CI: 11.05 to 12.94 kg, I^2^ = 98%) when including all groups. The intervention groups gained on average 11.60 kg (95% CI: 10.68 to 12.53 kg, I^2^ = 98%), while the control groups gained 12.44 kg (95% CI: 11.37 to 13.51 kg, I^2^ = 99%) (Table 2). Further, women gained on average 12.75 kg (95% CI: 11.69 to 13.81 kg) in studies including women of all BMI categories, 11.23 kg (95% CI: 9.81 to 12.66 kg) in studies including only women with OWOB, 11.93 kg (95% CI: 10.87 to 12.99 kg, I^2^ = 98%) in studies conducted in the West, and 12.19 kg (95% CI: 9.83 to 14.56 kg, I^2^ = 99%) the East (Table 2). Total GWG estimates were stratified based on whether self-reported pre-pregnancy weight (12.23 kg, 95% CI: 10.99 to 13.47 kg, I^2^ = 98%), or measured early pregnancy weight (11.72 kg, 95% CI: 10.21 to 13.23 kg, I^2^ = 98%) was used. Excluding one study that only reported GWG to mid-pregnancy (24 GW) [95], total mean GWG increased to 12.13 kg (95% CI: 11.17 to 13.09 kg). Among studies with less than 10 or 20% missing data, the estimate for total GWG was to 12.87 kg (95% CI 11.03 to 14.70 kg) and 11.69 kg (95% CI: 10.39 to 12.99 kg), respectively. Adjusting sample sizes of cluster RCTs, or excluding these did not change the estimated total GWG considerably (Supplemental Material S7: Sensitivity analyses of descriptive results).

### DRMA: effect of changes in EI and PA on GWG

In our assessment of the distribution of residuals, one study [91] led to large departures from linearity and normality of residuals, and thus, was excluded from all DRMA. Results of analyses of residuals and other goodness-of-fit statistics are presented in Supplemental Material  S9: Assumption testing.

**Fig. 4:**
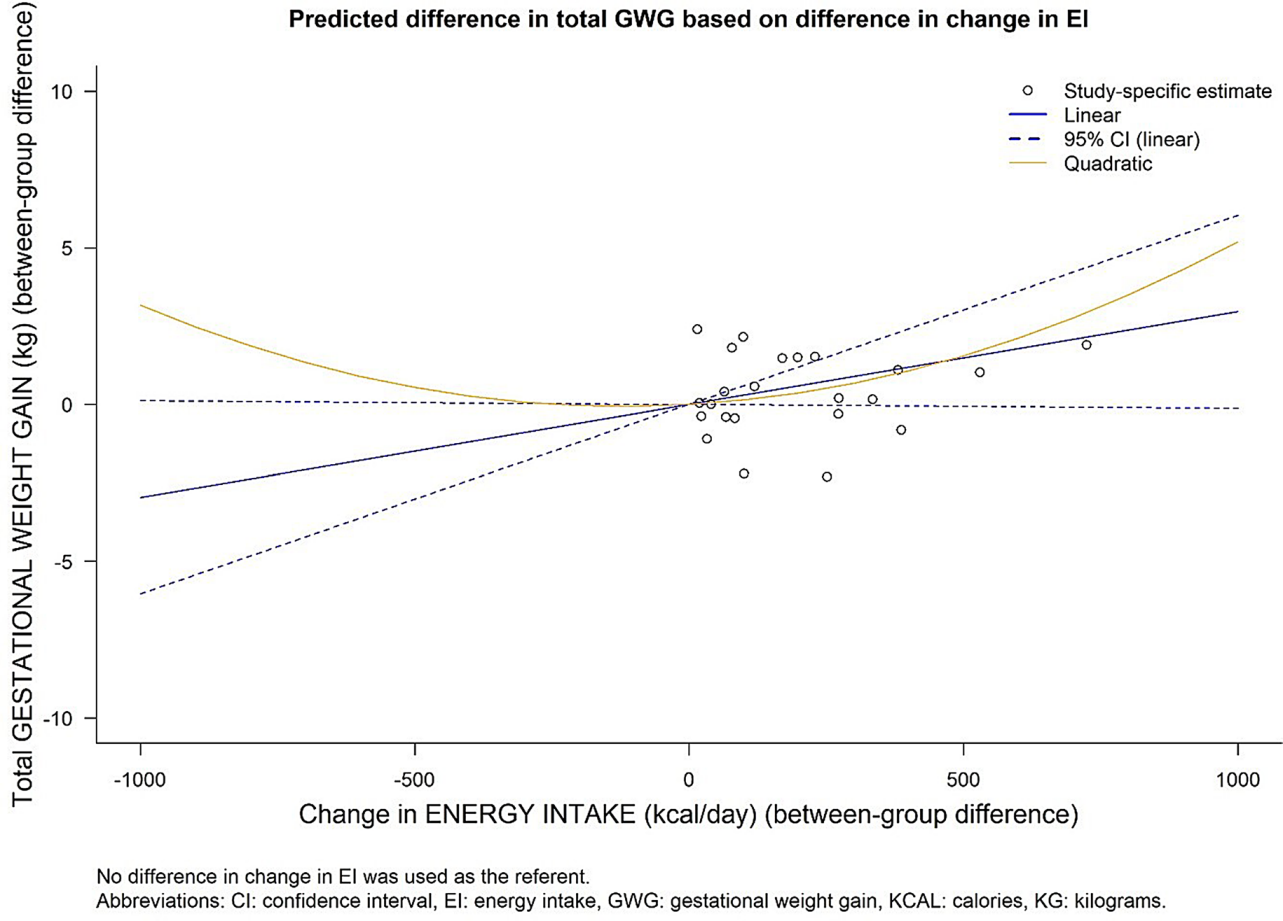
Predicted mean difference from linear and quadratic dose-response model: the association of EI with GWG

Results of the analyses of the DRMA between change in EI and GWG are presented in Table 3; Fig. 4. We estimated that every 100 kcal/day additional increase in EI corresponded to 0.30 kg more GWG (95% CI: -0.01 to 0.60, *p* = 0.06), in addition to the amount of total GWG that would have been gained without such an increase. Re-scaled to other potential recommended increases in EI by the third trimester, such as 250 kcal/ day or 500 kcal/day, this translates to 0.74 and 1.48 kg additional GWG, respectively.

**Table 3:** DRMA and subgroup analyses of the association between changes in EI and GWG

Main analyses	Predicted change in GWG (kg)	95% Confidence Interval	*P* value for EI	AIC
**Per 100 kcal/day increase in EI**				
Crude	0.30	-0.01 to 0.60	0.06	83.86
**Subgroup Analyses**	**Predicted change in GWG (kg)**	**95% Confidence Interval**	**P for subgroup difference**	**AIC**
*Pre-pregnancy / Early pregnancy BMI*				
All BMI ^a^	0.32	-0.09 to 0.74	0.85	85.82
Overweight / Obese	0.27	-0.18 to 0.71		
*Geographic location of study*				
East	-0.41	-0.96 to 0.15	0.005	78.60
West ^b^	0.46	0.22 to 0.69		
*Risk of bias in measurement of EI*				
High risk of bias	-0.20	-0.72 to 0.32	0.02	80.39
Low risk of bias	0.57	0.18 to 0.95		
*Missing data*				
< 10% missing	-0.05	-0.84 to 0.75	0.36	85.04
> 10% missing	0.36	0.01 to 0.71		
< 20% missing	0.27	-0.17 to 0.70	0.86	85.86
> 20% missing	0.32	-0.11 to 0.76		
*GWG assessment method*				
Self-reported pre-pregnancy weight	0.16	-0.20 to 0.51	0.19	84.20
Measured early pregnancy weight	0.54	0.09 to 0.99		

^a^ BMI was based on the inclusion/exclusion criteria of each study. One study included only women with normal BMI was grouped into the “All BMI” groups

^b^ A study conducted in Istanbul in Turkey was classified as “Western” [81]

Abbreviations: EI: energy intake, GWG: gestational weight gain, SMC: standardized mean change, AIC: Akaike Information Criterion

A non-linear relationship between change in EI and GWG during pregnancy was not indicated (P for quadratic term: 0.593) (Fig. 4). The between-study heterogeneity for the main analysis is presented in Supplement Material  S10, showing the VPC for EI across the exposure range. The proportion of variance attributable to heterogeneity varied from approximately 0.1–79% for changes in EI across the exposure range, suggesting a high portion of the variance in the data was influenced by between-study heterogeneity.

**Fig. 5:**
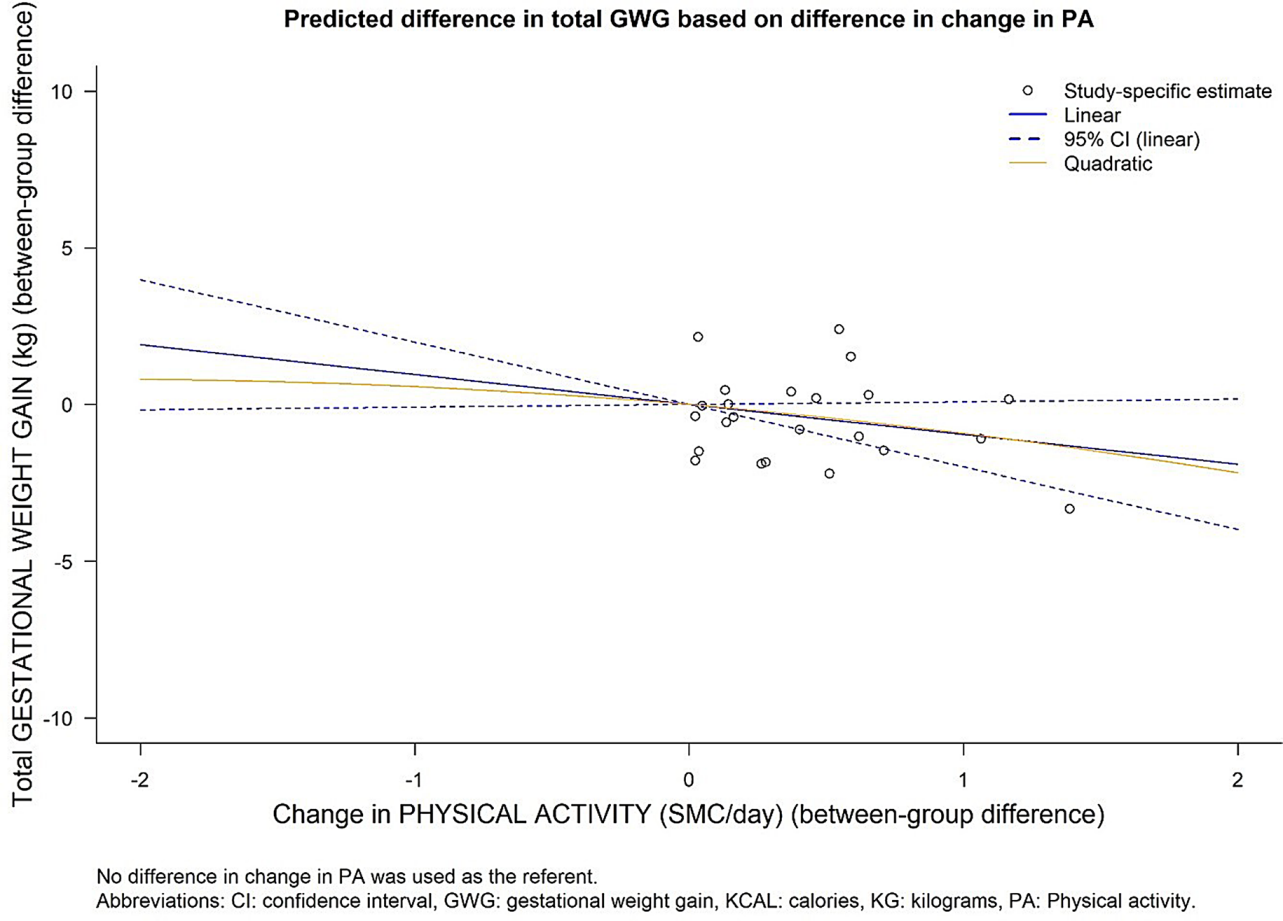
Predicted mean difference from linear and quadratic dose-response model: the association of PA with GWG

Results of the analyses of the DRMA between change in PA and GWG are presented in Supplemental Material S11, and Fig. 5. We estimated that every 0.25 SMC/day additional increase in PA corresponded to 0.24 kg less GWG (95% CI: -0.50 to 0.02, *p* = 0.07), than the amount of total GWG that would have been gained without such an increase. An increase of 0.25 SMC/day translates to about 5–15 min of moderate to vigorous PA, or about 100–120 kcal energy expenditure, increase per day. A non-linear relationship between change in PA and GWG during pregnancy was not indicated (P for quadratic term: 0.877) (Fig. 5).

The between-study heterogeneity for the main analysis is presented in Supplement Material S10, showing the VPC for PA across the exposure range. The proportion of variance attributable to heterogeneity varied from approximately 0.07–61% for changes in PA across the exposure range, suggesting a high portion of the variance in the data was influenced by between-study heterogeneity.

### Subgroup analyses

Subgroup analyses of the associations between change in EI and GWG were performed using the linear model and the results are presented in Table 3. Studies conducted in the West showed a significantly higher increase in GWG by 0.46 kg with every 100 kcal/day increase in EI (95% CI: 0.22 to 0.69 kg), while studies conducted in the East did not demonstrate a significant increase (-0.41 kg, 95% CI: -0.96 to 0.15) (p for subgroup difference: 0.005). Furthermore, studies with a low risk of bias (*n* = 12) in assessing EI showed a 0.57 kg increase in GWG (95% CI: 0.18 to 0.95 kg) per 100 kcal/ day increase in EI, while studies with a high risk of bias (*n* = 8) did not show any association (-0.20 kg, 95% CI: -0.72 to 0.32) (p for difference = 0.02). Comparison across BMI inclusion criteria, missing data categories, and method for measuring total GWG did not show any statistically significant differences between the groups.

Subgroup analyses of the associations between change in PA and GWG were performed using the linear model and the results are presented in Supplemental Material  S11. The only statistically significant subgroup differences were seen for studies with more vs. less than 10% missing data (studies with < 10% missing data: -0.79 kg, 95% CI: -1.20 to 0.38 kg, studies with > 10% missing data: -0.09 kg, -0.31 to 0.13 kg, p for difference: 0.004). However, a difference was not observed when stratifying the studies by whether or not they were missing 20% of data. In studies conducted among all women, a 0.14 kg reduction in GWG was predicted per 0.25 SMC/ day increase in PA (95% CI: -0.41 to 0.12), while in studies among women with OWOB, a 0.50 kg reduction was estimated (95% CI: -0.92 to -0.09, p for subgroup difference: 0.15). Comparison across geographic location of studies, risk of bias in assessing PA, and method for measuring total GWG did not show any statistically significant differences between the subgroups (Supplemental Material S11).

### Sensitivity analyses

Results excluding each study one-by-one (“leave-one-out analysis”) did not substantially deviate from those of the main analyses, with the predicted additional GWG per 100 kcal per day increase in EI ranging from 0.25 to 0.39 kg (Supplemental Material S12), compared to 0.30 kg as seen in the main analyses. For PA, the predicted additional GWG per 0.25 SMC per day increase in PA ranged from − 0.29 to -0.17 kg (Supplemental Material S12), compared to -0.24 kg in the main analyses. Additionally, we excluded two studies that assessed EI and PA only to mid-pregnancy (≤ 24 GW) [93, 95]. This exclusion did not yield any evidence of significant differences compared to the main analyses (Supplemental Material  S13). We also excluded studies that assessed GWG only to mid-pregnancy, which did not indicate any significant differences from the main analyses [95]. Adjusting sample sizes in cluster-RCTs, testing two different intra-class correlation coefficients, also did not change results compared to main analyses (Supplemental Material S13). Similarly, excluding the four cluster-RCTs did not significantly alter results. However, when testing different correlation coefficients for SMC in PA, the estimated effect size changed most notably to -0.13 kg, 95% CI: -0.39 to 0.13, *p* = 0.34), and − 0.36 kg, 95% CI: -0.73 to 0.02, *p* = 0.061) (Supplemental Material  S13).

## Discussion

In this systematical review of 21 RCT studies, we found that baseline EI before or in early pregnancy was on average 1977 kcal/day and increased by 132 kcal/day over the course of pregnancy. Women reduced their PA by 0.11 SMC/day by the third trimester. On average, women gained a total of 11.99 kg. Based on our DRMA, we identified a linear and positive association between increases in EI and GWG of borderline statistical significance (per 100 kcal/day increase: 0.30 kg, 95% CI: -0.01 to 0.60 kg, *p* = 0.06). A daily increase of 250 or 500 kcal in EI during pregnancy could therefore result in 0.74–1.48 kg additional GWG, respectively. Subgroup analyses indicated a stronger, positive association among studies conducted in the West compared to those in the East, and similarly in studies we considered at a low risk of bias in assessing EI. Sensitivity analyses, including testing alternate correlation coefficients for changes in EI, as well as leave-one-out analyses, suggested our results to be robust. For PA, our DRMA indicated a linear, inverse association between increases in PA and GWG (-0.24 kg, 95% CI: -0.50 to 0.02, *p* = 0.07). Subgroup analyses indicated that effects may be more pronounced among women with OWOB, but results did not reach statistical significance. However, while sensitivity analyses generally did not indicate important differences, using alternative correlation coefficients for estimating the SMC in PA did somewhat alter our results, as did stratifying studies based on the amount of missing data. The sum of the evidence therefore suggests that increases in EI and reductions in PA, as observed through randomized lifestyle intervention trials, may lead to higher GWG. Our descriptive analysis suggests average increases in EI and GWG very similar compared to a previous meta-analysis from 2016, which suggested increases of 114 kcal/ day on average, and 12.00 kg total GWG, with a correlation coefficient (r) = 0.32 (*p* = 0.11) based on meta-analytical means of study-level averages from 18 studies [44]. However, Jebeile et al. only investigated the association between changes in EI and GWG using study-level values to assess correlations, while we used a DRMA to pool within-study estimates for the changes in GWG associated with changes in EI. This approach has considerable advantages over assessing the association only at the study-level, most importantly, ecological fallacies are avoided. Therefore, study-level differences in EI or GWG (e.g., different dietary habits in different countries) may not have affected our results.

Our subgroup analyses found a significant positive association between EI and GWG among studies conducted in high-income countries in the West (in North America, Europe, and Australia). Consistent with our findings, a systematic review of 12 observational studies in high-income countries suggested lower energy intake might reduce GWG in pregnancy [103]. In contrast, another systematic review reported that findings regarding the association between EI and GWG did not differ between low-, middle-, and high-income countries [104]. When stratifying by risk of bias, high-quality (i.e., low risk of bias) studies yielded statistically significant results rather than low-quality (i.e., high risk of bias) studies. Our findings were therefore also aligned with the results from high-quality studies from the previous systematic review [104]. In our analyses, high-quality studies were characterized by the use of 24-hour dietary recall or similar assessments, rather than FFQ. In previous studies, it was found that the correlation coefficient between the reported energy intake based on a FFQ, and the actual energy intake of provided experimental diets was 0.74 among women, and individually reported energy intake ranged from 56.3 to 159.6% as a percentage of actual intake [105]. Large inter-individual differences in the accuracy of FFQ for estimating EI may therefore affect results, and this is one explanation for why our results differed considerably when excluding studies using FFQ.

Findings from other studies regarding changes in PA and GWG are more sparse: A recent umbrella review showed that regular participation in moderate-intensity PA may reduce the risk of excessive GWG [106]. However, the impact of changes in PA on GWG was not quantified. A recent meta-analysis of 32 prospective and objective studies found that there were significant increases in resting energy expenditure (REE) and total energy expenditure (TEE) throughout pregnancy, however the extent of the increase was highly variable among studies, and inconclusive in relation to GWG [107]. Although the link between PA and GWG was not established, another systematic review of 12 randomized controlled trials (RCT) intervening on PA found less GWG in the intervention groups, compared with the control groups [108]. Altogether, while results for changes in PA failed to reach statistical significance, a negative trend was observed, and PA has been shown to be protective against excessive GWG based on previous studies, and additional benefits and the safety of PA during pregnancy have been established [38, 106].

Results from the DRMA for both EI and PA were highly heterogeneous across subgroup and sensitivity analyses. For example, per 100 kcal/day increase in EI resulted in 0.57 kg change in total GWG in low risk of bias studies, while the same increment in EI led to -0.20 kg change in total GWG in high risk of bias studies. Subgroup and sensitivity analyses were likely affected by low statistical power and uncontrolled confounding by other study-level variables, and results should therefore be interpreted cautiously. High heterogeneity between studies may obscure the true effect size, as discussed further below. Therefore, rather than providing exact quantification of the association between changes in EI and PA with GWG, our results are useful in demonstrating that (1) GWG is modifiable through changes in EI and PA, and (2) higher increases in EI (≥ 340 kcal/ day in 2nd trimester, ≥ 452 kcal/ day in 3rd trimester) [31], or larger decreases in PA could be a considerable risk factor for excessive GWG, especially among women with OWOB. Per our analyses, a 452 kcal/day increase by the third trimester, in line with current recommendations, leads to 1.34 kg higher total GWG (95% CI: -0.05 to 2.73 kg), or if we restrict our analysis to studies using more reliable EI assessment methods, to 2.56 kg higher GWG (95% CI: 0.83 to 4.28 kg). Further, we observed that numerous studies reported lower than recommended increases in caloric intake by the 3rd trimester (Fig. 3A), as well as decreases in PA levels between early and late pregnancy (Supplemental Material S8), while multiple studies conducted among women with OWOB reported mean total GWG above the IOM’s recommended range of up to 11 kg for women with overweight (Fig. 3B).

To our knowledge, this is the first DRMA to quantify the association of EI and PA with GWG, based on evidence from RCTs. By using data from RCTs, the impact of confounding variables (e.g., maternal age or socioeconomic status) on the association of EI and PA with GWG was mitigated. Post-randomization imbalances may still have been present in some variables, such as sleep, although we utilized the randomized study designs to analyze these associations within studies. Prior research suggests that shorter sleep duration and higher frequency of awakening were associated with a decrease in PA and an increase in GWG in pregnant women with OW/OB [109]. However, without a larger amount of data, additional information on potential confounding variables, and their distribution across study groups, it is not possible to predict the direction of confounding. Still, we did not identify any potential biases associated with the randomization procedures, and therefore do not consider confounding a major threat to the validity of our analyses. By incorporating data from diet and PA intervention trials, the exposure range for changes in EI and PA was possibly larger than what would have been reported in an observational study. Strengths and limitations of using the SMC are outlined in the supplement (Supplemental Material S14: Strengths and limitations of using SMC for PA). In addition, using change in EI and PA allowed us to partially account for baseline differences in EI and PA, which could otherwise confound analyses if we solely relied on the final values. Finally, by using the RMC/ SMC approach, rather than comparing EI/ PA across two different groups, each study group served as their own control, reducing the error term and increasing the precision.

On the other hand, while our approach using within-study differences in group means is a clear improvement over simply using between-study differences in study means to explore this association, our analyses are not entirely protected from aggregation bias, due to the use of group means in EI and PA, rather than individual level data. Also, repeated measurements of EI and PA may be affected by regression to the mean, where no true changes were measured, but fluctuations in measurement validity or accuracy [110]. An important limitation of the body of evidence is that 14 of 21 included RCTs were assessed at a high risk of bias, primarily due to the use of less accurate assessment methods, such as FFQs for EI, or self-reported PA measures, and missing data. This likely introduced non-differential exposure misclassification, biasing effect estimates towards the null and causing substantial heterogeneity in our meta-analyses. Our subgroup analyses limiting to RCTs with more accurate exposure assessments yielded stronger and more precise estimates between EI and GWG, while RCTs using lower-quality methods yielded null associations with wider confidence intervals. For PA, results did not differ by assessment method. Regarding missing data, we conservatively assigned a high risk of bias to studies with more than 20% of exposure or outcome data missing, given uncertainty about whether missingness depended on true values. One sensitivity analysis showed a stronger effect of PA in reducing total GWG among studies with very low attrition (< 10%), suggesting that missing data may have attenuated associations. Overall, limitations in exposure assessment and missing data likely led to an underestimation of the true effect size and should be considered when interpreting our results. Further, studies used numerous methods for assessing total GWG, using measurements from different GW in early and later pregnancy, and relying on self-reported or measured values of baseline weights. The small within-study differences together with the large between-study differences in GWG highlighted by Fig. 3B demonstrate that these methodological differences gave rise to high between-study heterogeneity in GWG estimates. Within-study estimates of the association of EI, PA, and GWG as utilized in the DRMA would not be affected by this, although the heterogeneity observed in both descriptive and inferential analyses could have been affected by the GWG assessment. Heterogeneity may also have arisen from other sources, in addition to measurement errors. Diet and exercise patterns may vary across countries and cultures in important ways besides caloric intake and energy expenditure, including macronutrient composition, food quality, or work-based vs. leisure-time PA [111]. Other factors may include healthcare system differences, including the standard prenatal care received by women. These contextual differences likely contributed to the high heterogeneity that we observed. In addition, we did not identify a sufficient number of studies reporting data that could be used to assess trimester-specific associations, because most included RCTs only reported EI, PA, and/or GWG at two time points (i.e., baseline and late pregnancy/ at birth). As previously mentioned, pregnant women are encouraged to increase EI by 340 kcal and about 450 kcal per day in 2nd and 3rd trimesters, respectively, compared to 0 kcal/day increase in the 1st trimester [30, 31]. Recent meta-analyses also suggest that absolute EI is highest in the 3rd trimester, compared to earlier pregnancy stages [112]. Therefore, the average increase of 132 kcal/day throughout pregnancy that we reported does not reflect the variations in EI change from early to late pregnancy. Future studies should focus on trimester-specific data to refine analyses of the associations between changes in EI, PA, and GWG. Finally, our analyses were limited to exploring the univariable associations of changes in EI and PA with total GWG, respectively, and we were therefore unable to explore the complex relationship of changes in both EI and PA with GWG concurrently. In terms of generalizability, because studies conducted solely among underweight women were excluded from our review, our findings are not applicable to populations where maternal undernutrition is prevalent.

Given the limitations discussed above, our findings exhibiting borderline statistical significance, should be interpreted cautiously. While precise quantification of the association between changes in EI/PA and GWG was not possible with the available data, our results are useful in demonstrating that GWG is modifiable through changes in EI and PA during pregnancy, and that larger increases in EI or decreases in PA may lead to higher GWG. In addition, our study emphasizes the need for more individualized nutrition and PA recommendations, based on pre-pregnancy BMI and existing lifestyle, rather than uniform guidelines for all pregnant populations.

## Electronic supplementary material

Below is the link to the electronic supplementary material.

Supplementary Material 1

## Data Availability

Data described in the manuscript and analytic code will be made available upon request.

## Abbreviations

GWG: Gestational Weight Gain
LGA: large for gestational age
IOM: Institute of Medicine
BMI: Body Mass Index
EI: Energy Intake
WHO: World Health Organization
ACOG: American College of Obstetricians and Gynecology
USDA: US Department of Agriculture
CDC: Centers for Disease Control
PA: Physical Activity
DRMA: Dose-Response Meta-Analysis
PRISMA: Preferred Reporting Items for Systematic Reviews and Meta-Analyses
CENTRAL: Cochrane Central Register of Controlled Trials
RCT: Randomized Controlled Trial
FFQ: Food Frequency Questionnaires
RMC: Raw Mean Change
SMC: Standardized Mean Change
VPC: Variance Partition Coefficient
OWOB: Overweight or Obesity
GW: Gestational Weeks
NW: Normal Weight
