# Supplementary material for: Energy intake and physical activity over the course of pregnancy and gestational weight gain: a systematic review and dose-response meta-analysis of data from randomized controlled lifestyle intervention trials
Source: Nutr J. 2025 Aug 7;24:123. doi: 10.1186/s12937-025-01182-w (PMC12333096; doi:10.1186/s12937-025-01182-w)
Supplement: Supplementary file 1 — Supplementary Material 1 [file 12937_2025_1182_MOESM1_ESM.pdf]

Supplemental Material S1: References identified during preliminary scoping

29 primary studies and 4 systematic reviews were identified.[1-33]

Supplemental Material S2: Full electronic search strategies

#	Search String	Hits	Comment
**PUBMED (https://pubmed.ncbi.nlm.nih.gov/advanced/)**			
#1	Pregnancy [Mesh] OR pregnan* [tiab] OR gestation* [tiab] OR prenatal [tiab] OR antenatal [tiab])	1,127,031	
#2	("Prenatal Nutritional Physiological Phenomena" [Mesh] OR energy intake [Mesh] OR energy intake [tiab] OR energy requirement* [tiab] OR calori* [tiab] OR kilojoule* [tiab] OR kcal [tiab] OR food* [tiab] OR nutrition* [tiab] OR nutritional requirements [Mesh] OR eating [Mesh] OR eating [tiab] OR diet [Mesh] OR diet* [tiab])	1,419,405	
#3	(weight gain [Mesh] OR weight gain* [tiab] OR weight increase* [tiab] OR weight change* [tiab] OR gestational weight gain [Mesh] OR gestational weight gain* [tiab] OR gwg [tiab] OR fat mass [tiab])	115,827	
#4	Animals [Mesh] NOT Humans [Mesh]	4,816,776	
#5	#1 AND #2 AND #3	6,545	
#6	#5 NOT #4	4,162	
#7	Review* [ti] OR meta-analysis [ti] OR comment* [ti] OR review [pt] OR systematic review [pt] OR meta-analysis [pt] OR comment [pt] OR systematic review [Filter] OR meta-analysis [Filter]	4,010,035	
#8	#6 NOT #7	3,359	
**Web of Science (http://apps.webofknowledge.com/WOS_AdvancedSearch_input.do?SID=C2cL6kxjcFWVmrFNACI&product=WOS&search_mode=AdvancedSearch)**			
#1	TS = (pregnan* OR gestation* OR prenatal OR antenatal)	680,711	
#2	TS = ("energy intake" OR "energy requirement*" OR calori* OR kilojoule* OR kcal OR food* OR nutrition* OR eating OR diet*)	2,189,342	
#3	TS = ("weight gain*" OR "weight increase*" OR "weight change*" OR "gestational weight gain*" OR gwg OR "fat mass")	130,189	
#4	(TI = animal* OR AK = animal* OR KP= animal* OR TI = mouse OR AK = mouse OR KP = mouse OR SU= veterinary sciences OR SU= zoology) NOT (TI = human* OR AK = human* OR KP= human* OR TI = wom?n OR AK = wom?n OR KP = wom?n OR SU=Obstetrics & Gynecology)	2,471,010	
#5	#1 AND #2 AND #3	5,820	
#6	#5 NOT #4	5,163	

#7	(TI= review* OR AK= review* OR KP= review* OR TI= systematic review OR AK= systematic review OR KP= systematic review OR TI= "meta-analysis" OR AK= “meta-analysis” OR KP= “meta-analysis” OR TI=comment* OR AK= comment* OR KP= comment*)	1,043,697	
#8	#6 NOT #7	4,896	
**PubAg (https://pubag.nal.usda.gov/advanced)**			
#1	All fields: pregnancy OR gestation OR prenatal OR antenatal	21,361	No Mesh terms in PubAg. No truncation.
#2	All fields: "energy intake" OR "energy requirement" OR calorie OR Kilojoule OR Kcal OR food OR Nutrition OR Eating OR diet	528,642	
#3	All fields: “weight gain” OR “weight increase” OR “weight change” OR “gestational weight gain” OR gwg OR “fat mass”	21,733	
#4	#1 AND #2 AND #3	955	Excluding animal studies or reviews not feasible
**EMBASE (https://www.embase.com/#advancedSearch/default)**			
#	Search String	Hits	Comment
#1	('pregnancy'/de OR 'first trimester pregnancy'/de OR 'second trimester pregnancy'/de OR 'third trimester pregnancy'/de OR pregnan*:ti,ab,kw OR gestation*:ti,ab,kw OR prenatal:ti,ab,kw OR antenatal:ti,ab,kw)	1,239,690	
#2	('maternal nutrition'/de OR 'caloric intake'/de OR “energy intake”:ti,ab,kw OR “energy requirement*”:ti,ab,kw OR calori*:ti,ab,kw OR kilojoule*:ti,ab,kw OR kcal:ti,ab,kw OR food*:ti,ab,kw OR 'nutrition'/exp OR 'nutritional requirement'/de OR nutrition*:ti,ab,kw OR 'eating'/de OR eating:ti,ab,kw OR 'diet'/exp OR diet*:ti,ab,kw)	3,181,805	Quotation marks needed for phrase searching
#3	('body weight gain'/exp OR “weight gain*”:ti,ab,kw OR “weight increase*”:ti,ab,kw OR “weight change*”:ti,ab,kw OR 'gestational weight gain'/exp OR “gestational weight gain*”:ti,ab,kw OR gwg:ti,ab,kw OR “fat mass”:ti,ab,kw)	195,314	
#4	'Animals'/exp NOT 'Humans'/exp	5,841,671	
#5	review:ti OR 'meta-analysis':ti OR comment*:ti OR review:it OR	3,376,7	

	'systematic review':it OR 'meta analysis':it OR comment:it OR 'systematic review'/exp OR 'meta analysis topic'/exp	98	
#6	#1 AND #2 AND #3	11,494	
#7	#6 NOT #4	8,239	
#8	#7 NOT #5	6,953	
**CENTRAL (https://www.cochranelibrary.com/advanced-search?q=*&t=6)**			
#1	Mesh descriptor: [Pregnancy] this term only OR pregnan*:ti,ab,kw OR gestation*:ti,ab,kw OR prenatal:ti,ab,kw OR antenatal:ti,ab,kw	78,186	
#2	Mesh descriptor: [Prenatal Nutritional Physiological Phenomena] explode all trees OR Mesh descriptor: [Energy intake] this term only OR Mesh descriptor: [Nutritional requirements] this term only OR Mesh descriptor: [Eating] this terms only OR Mesh descriptor [Diet] explode all trees OR "energy intake":ti,ab,kw OR energy requirement*:ti,ab,kw OR calori*:ti,ab,kw OR kilojoule*:ti,ab,kw OR kcal:ti,ab,kw OR food*:ti,ab,kw OR nutrition*:ti,ab,kw OR eating:ti,ab,kw OR diet*:ti,ab,kw	151,283	No quotation marks when truncating
#3	Mesh descriptor: [Weight gain] explode all trees OR Mesh descriptor: [gestational weight gain] explode all trees OR weight gain*:ti,ab,kw OR weight increase*:ti,ab,kw OR weight change*:ti,ab,kw OR gestational weight gain*:ti,ab,kw OR gwg:ti,ab,kw OR fat mass:ti,ab,kw	75,710	
#4	Animal:ti,kw NOT humans: ti,kw	6,908	
#5	#1 AND #2 AND #3	3,113	
#6	#5 NOT #4	3,077	
#7	Select “Trials” tab	2,924	
**CINAHL (http://web.a.ebscohost.com/ehost/search/advanced?vid=0&sid=ad1e25cd-979d-46cf-b072-40dacba42676%40sessionmgr4006)**			
#1	((MH "Pregnancy") OR (TI pregnan* OR AB pregnan*) OR (TI gestation* OR AB gestation*) OR (TI prenatal OR AB prenatal) OR (TI antenatal OR AB antenatal))	280,334	
#2	((MH "Maternal Nutritional Physiology+") OR (MH "Prenatal Nutritional Physiology") OR (MH "energy intake") OR TI “energy intake” OR AB “energy intake” OR TI energy requirement* OR AB energy requirement* OR TI calori* OR AB calori* OR TI kilojoule* OR AB kilojoule* OR TI kcal OR AB kcal OR TI food* OR AB food* OR (MH "nutritional requirements") OR (MH “nutrition”) OR TI nutrition* OR AB nutrition* OR (MH "Eating Behavior+") OR TI eating OR AB eating OR (MH "diet+") OR TI diet* OR AB diet*)	381,266	Truncation and quotation marks don’t mix in CINAHL
#3	((MH "Weight Gain+") OR TI weight gain* OR AB weight gain* OR TI weight increase* OR AB weight increase* OR TI weight change*	134,382	

	OR AB weight change* OR (MH "Gestational Weight Gain") OR TI gestational weight gain* OR AB gestational weight gain* OR TI gwg OR AB gwg OR TI “fat mass” OR AB “fat mass”)		
#4	(MH "Animals+" OR TI animal* OR SU animal*) NOT (MH "Humans" OR TI human* OR SU human*)	190,409	
#5	(TI Review* OR TI "meta-analysis" OR TI comment* OR PT review OR PT "systematic review" OR PT "meta-analysis" OR PT comment*)	959,562	
#6	#1 AND #2 AND #3	3,816	
#7	#6 NOT #4	3,497	
#8	#7 NOT #5	2,871	

All searches conducted between 04/23/2021 and 04/26/2021 by Sophie Michel.

Supplemental Material S3

PECOS[1] Selection Criteria

Population	Pregnant women with singleton pregnancies, pre-pregnancy BMI category reported
Exposure	EI and PA reported as a continuous variable, for at least two time points
Comparison	Exposure and outcome data reported separately for at least two study groups
Outcome	GWG (reported as a continuous variable)
Study design	Randomized controlled trials
Setting	All settings and geographic locations were considered
Timeframe	No criteria regarding the study period or publication date were used
Languages	No restrictions

Note:

1 PECOS: Population, Exposure, Comparison, Outcome, and Study design

Abbreviations: PA: Physical activity, EI: energy intake, GWG: gestational weight gain

Supplemental Material S4

Table of Cochrane's ROB 2 tool domains modified using ROBINS-E tool components

**Risk of bias domain**	**Modification**	**Explanation**
Randomization process	No	N/A
Timing of participant identification/ recruitment (cluster-RCTs only)	No	N/A
Measurement of the exposure	Yes	Replaces "Domain 2: Deviations from the intended interventions (effect of assignment to/ adhering to intervention)"
Measurement of the outcome	No	N/A
Missing data	Yes	Modified to include assessments of both missing outcome and missing exposure data
Selection of the reported result	Yes	Modified to probe selection of results based on both different outcome and exposure assessment methods.
Overall risk of bias	No	N/A

Supplemental Material S5: Statistical analyses

Data preparation

Prior to meta-analysis, standard deviations were derived from 95% CIs, as needed.[34] Where only medians for PA were reported, means were estimated using the method for unknown non-normal distributions.[35]

Raw mean change (RMC) in EI and standardized mean change (SMC) in PA during pregnancy and corresponding variances were estimated using the raw mean values and variances in each study group at the earliest (T1) and latest assessed time points (T2), and combined with correlation coefficients applied to these repeated measurements, (0.60 and 0.48 were used for EI, and 0.75 and 0.76 were used for PA) based on studies for which we had access to the required data,[36,37] and previous meta-analyses.[12] However, we conducted sensitivity analyses testing various different correlation coefficients ranging from 0.35 to 0.76.

Assumptions

To test the assumptions of linear mixed models regarding linearity and the distribution of errors, histograms and Q-Q plots were used to assess the distribution of residuals, and the dose levels vs. the de-correlated residuals were plotted.[38-40]

Data analysis: Main analyses: Association of EI and PA with GWG

Dose-response meta-analysis can be used to pool dose-response associations from aggregate data of multiple studies, where estimates are reported for different dose levels, using one common referent group.[41] Greenland and Longnecker developed an approach for modeling linear trends from aggregate dose-response data using generalized least-square regression, while back-estimating the covariance between the outcomes at different dose levels, as different dose levels from one study are correlated due to the common referent group.[42] Berlin et al. proposed a meta-analysis method that applies a random effect regression model to multiple dose-response studies.[43]

Crippa et al. further developed these methods for differences of means of continuous outcomes,[44] and also an extension of the one-stage approach that allows for the estimation of models with additional parameters beyond those involved in simple linear models, even when some included studies do not report the necessary number of non-referent observations, described further below.[38] The *dosresmeta* package and publication of worked examples make dose-response meta-analyses accessible to a wide range of users.[38,44-46]

For all studies, the lowest “dose” level (i.e., the largest decrease or smallest increase in EI/PA from early pregnancy) was treated as the referent group.

The method of maximum likelihood (ML) was used to estimate the random-effects model, which allows for goodness-of-fit comparisons of different models using the Akaike information criterion (AIC).[38,47]

Adjusting cluster-RCT sample sizes in sensitivity analyses:

Sample sizes of cluster-RCTs were adjusted using the formula:[48]

$$\frac{N}{1+(M-1)*ICC}$$

N is the original sample size; M is the average cluster size; and ICC is the intraclass correlation coefficient. The ICC was obtained for each study, if reported, and otherwise borrowed from another study.[48] We used the ICC from the study reporting the largest ICC,[49] and sensitivity analyses using the ICC from another study were undertaken.[50]

Supplemental Material S6: Individual risk of bias assessment results

		Guelinckx, I., et al., 2010	Luoto, R., et al., 2011	Hui, A. L., et al., 2012	Rauh, K., et al., 2013	Dodd, J. M., et al., 2014	Hui, A. L., et al., 2014	Jing et al., 2015	Poston, L., et al., 2015	Asci et al., 2016	Smith, K., et al., 2016	Chan et al., 2018	Downs et al., 2021	Buckingham-Schutt et al., 2019	Phelan et al., 2018+2019	Dodd et al., 2019	Gunther et al., 2019	Haijian et al., 2020	Ding B et al., 2020	Huang et al., 2020	Ferrara, A., et al., 2020	Liu et al., 2021
cluster-randomized trial	Risk of bias arising from the randomization process		low		low												low	low				
	Risk of bias arising from the timing of identification or recruitment of participants in a cluster-randomized trial		low		high												low	low				
	Risk of bias in measurement of the exposure		high		some concerns												high	high				
	Risk of bias in measurement of the outcome		low		low												low	low				
	Risk of bias due to missing data		high		high												some concerns	low				
	Risk of bias in selection of the reported result		low		low												low	low				
	Overall risk of bias		high		high												high	high				
individual randomized trial	Risk of bias arising from the randomization process	low		low		low	low	low	low	low	low	low	low	low	low	low			low	low	low	low
	Risk of bias in measurement of the exposure	some concerns		some concerns		high	some concerns	high	high	some concerns	low	some concerns	some concerns	low	some concerns	high			some concerns	some concerns	low	low
	Risk of bias in measurement of the outcome	low		low		low	low	low	low	low	low	low	low	low	low	low			low	low	low	low
	Missing data	high		high		high	low	some concerns	high	some concerns	high	high	some concerns	some concerns	low	low			low	high	some concerns	high
	Risk of bias in selection of the reported result	low		low		low	low	low	low	low	low	low	low	low	low	low			low	low	low	low
	Overall risk of bias	high		high		high	some concerns	high	high	some concerns	high	high	some concerns	some concerns	some concerns	high			some concerns	high	some concerns	high

Supplemental Material S7: Sensitivity analyses of descriptive results

Descriptive analyses of changes in EI	Different correlation coefficients for estimating variance of RMC:	1 (original): 132 kcal, 95% CI: 54 to 209 kcal, I^2= 100%<br><br>2: 132 kcal, 95% CI: 55 to 209 kcal, I^2= 100%<br><br>3: 132 kcal, 95% CI: 55 to 209 kcal, I^2= 100%<br><br>4: 132 kcal, 95% CI: 54 to 210 kcal, I^2= 100%
	10% Missing data	Studies with less than 10% missing data: 96 kcal, 95% CI: -16 to 209 kcal, I^2= 100%<br><br>Studies with more than 10% missing data: 144 kcal, 95% CI: 46 to 241 kcal, I^2= 100%
	20% Missing data	Studies with less than 20% missing data: 171 kcal, 95% CI: 57 to 285 kcal, I^2= 100%<br><br>Studies with more than 20% missing data: 71 kcal, 95% CI: -9 to 151 kcal, I^2= 100%
	Risk of bias	High risk of bias: 159 kcal, 95% CI: -27 to 344 kcal, I^2= 100%
		Low risk of bias: 111 kcal, 95% CI: 58 to 165 kcal, I^2= 100%
	Timing of dietary assessment	Excluding two studies that only reported changes in EI to mid-pregnancy (< = 24 GW):[51,52]<br><br>118 kcal/day, 95% CI: 37 to 199 kcal/ day<br><br>Excluding two studies that did not report until exactly when EI was assessed: [53,54]<br><br>123 kcal/day, 95% CI: 31 to 216 kcal/day.
Descriptive analyses of changes in PA	Different correlation coefficients for estimating SMCC:	1: (original): -0.11 SMCC/day, 95% CI: -0.233 to 0.12, I^2= 100%<br><br>2: -0.06 SMCC/day, 95% CI: -0.26 to 0.14, I^2= 100%<br><br>3: -0.11 SMCC/day, 95% CI: -0.30 to 0.07, I^2= 100%

		4: -0.07 SMCC/day, 95% CI: -0.22 to 0.08, I^2= 100%
	10% missing data	Studies with less than 10% missing: -0.01 SMCC/day (95% CI: -0.55 to 0.53, I^2= 100%)<br><br>Studies with more than 10% missing: -0.14 SMCC/day (95% CI: -0.38 to 0.10, I^2= 100%)
	20% missing data	Studies with less than 20% missing: 0.05 SMCC/day (95% CI: -0.30 to 0.39, I^2= 100%)<br><br>Studies with more than 20% missing: -0.31 SMCC/day (95% CI: -0.52 to -0.10, I^2= 100%)
	Risk of bias	High risk of bias: -0.16 SMCC/day (95% CI: -0.44 to 0.12, I^2= 100%
		Low risk of bias: -0.02 SMCC/day (95% CI: -0.42 to 0.39, I^2= 100%)
	Timing of PA assessment	Excluding two studies that only reported changes in PA to mid-pregnancy (< = 24 GW)[51,52]:<br><br>-0.12 SMCC/day, 95% CI: -0.37 to 0.13.<br><br>Excluding two studies that did not report until exactly when PA was assessed<br><br>-0.14 kcal/day, 95% CI: -0.42 to 0.14.[53,54]
Descriptive analyses of total GWG	Addressing cluster-RCTs	Adjusting the sample sizes of cluster-RCT study groups using the ICC from the study reporting the largest ICC[49]:<br><br>11.97 kg, 95% CI: 11.03 to 12.91 kg.<br><br>Using the ICC from another study[50]:<br><br>11.99 kg, 95% CI: 11.04 to 12.93 kg.<br><br>Excluding cluster-RCTs:<br><br>11.37 kg, 95% CI: 10.42 to 12.32.
	Timing of GWG assessment	Excluding one study that only reported GWG to mid-pregnancy (24 GW)[51]:<br><br>12.13 kg, 95% CI: 11.17 to 13.09 kg.

Supplemental Material S8: Forest plot of changes in PA (SMCC/day)

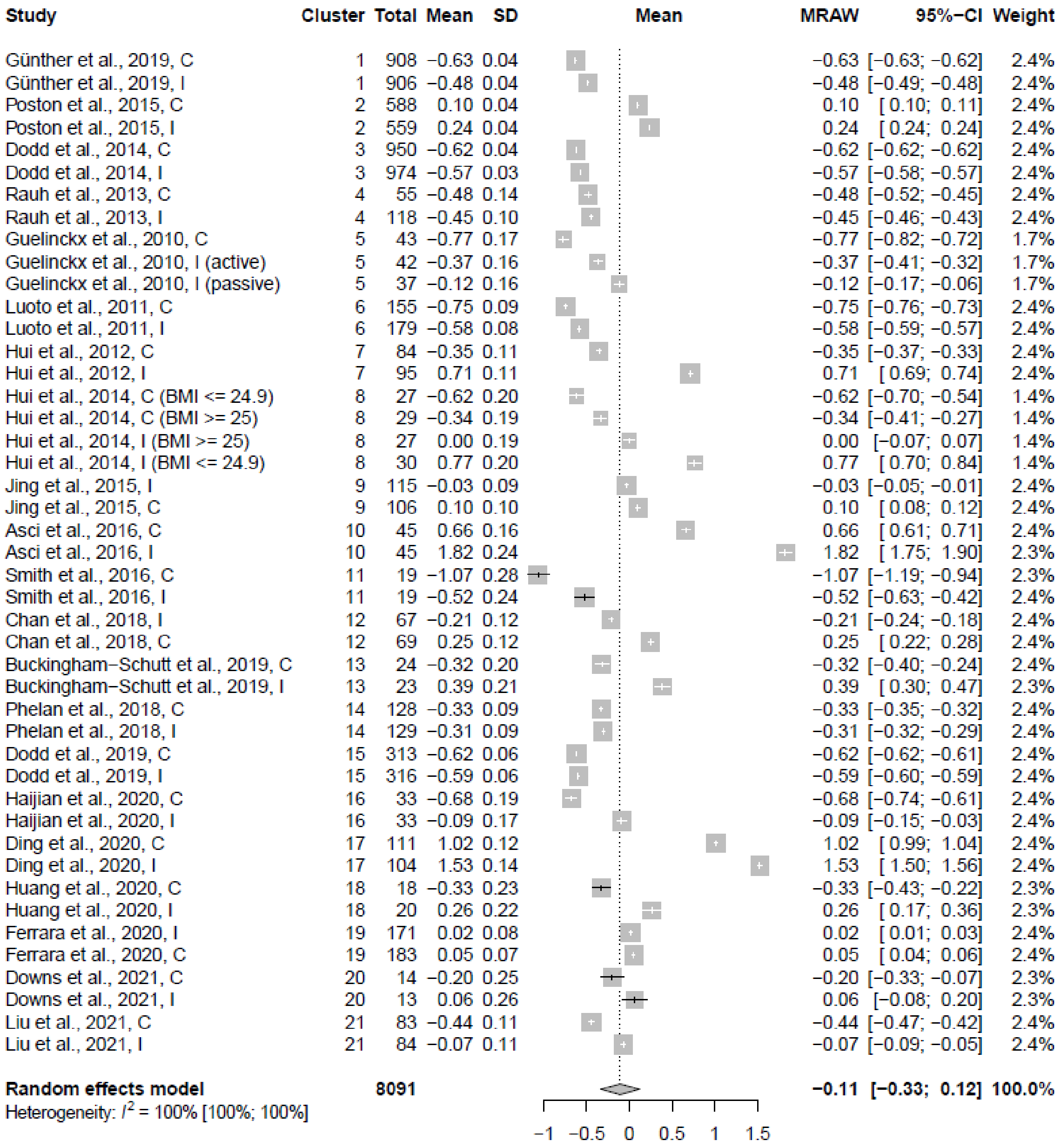

Figure. Pooled change in PA (SMCC) across studies (per day), accounting for clustering of study groups within studies (2-4 groups per study) via three-level meta-analysis. Abbreviations: CI: confidence interval**,** SD: standard deviation, SMC: standardized mean change

Supplemental Material S9: Assumptions testing for DRMA, which lead to the exclusion of one study from the main analyses.[55]

DRMA of change in EI and total GWG:

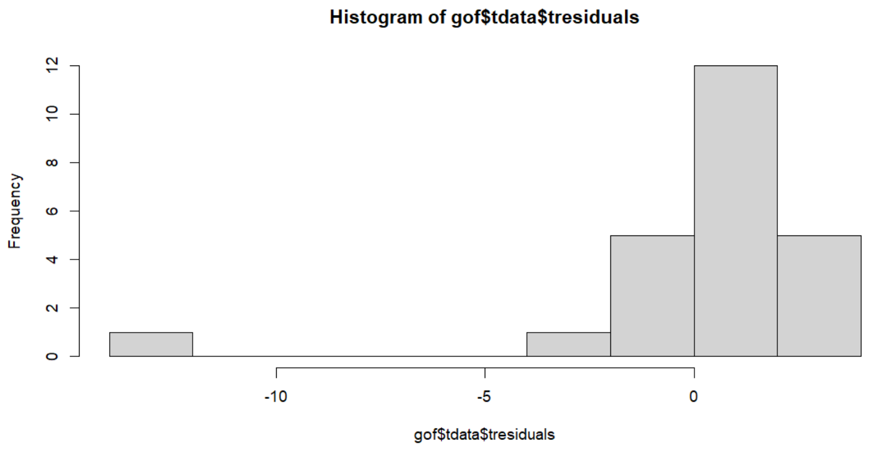

Figure 1: Histogram of residuals

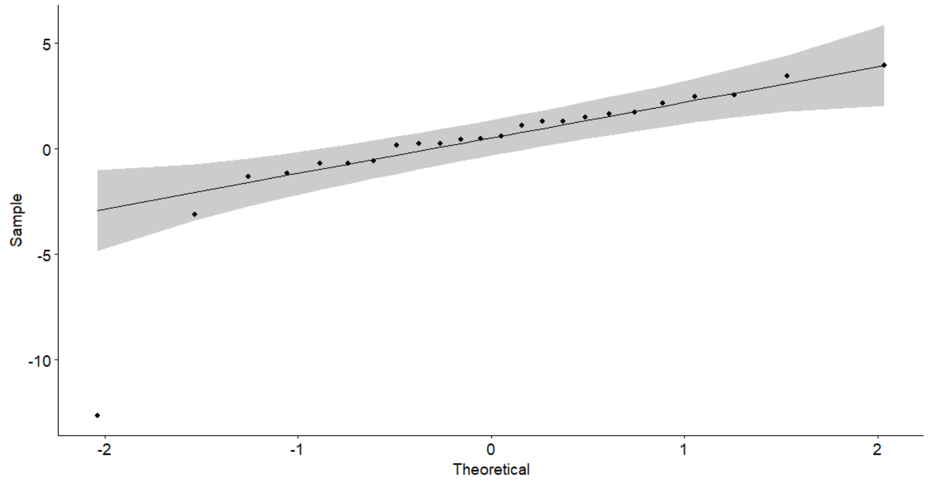

Figure 2: QQ plot of residuals

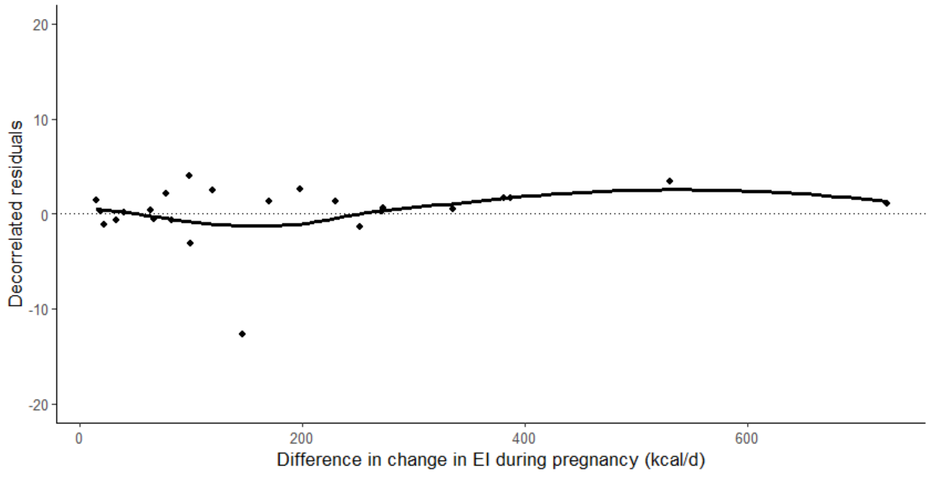

Figure 3: De-correlated residuals vs. exposure plot

DRMA of change in PA and total GWG:

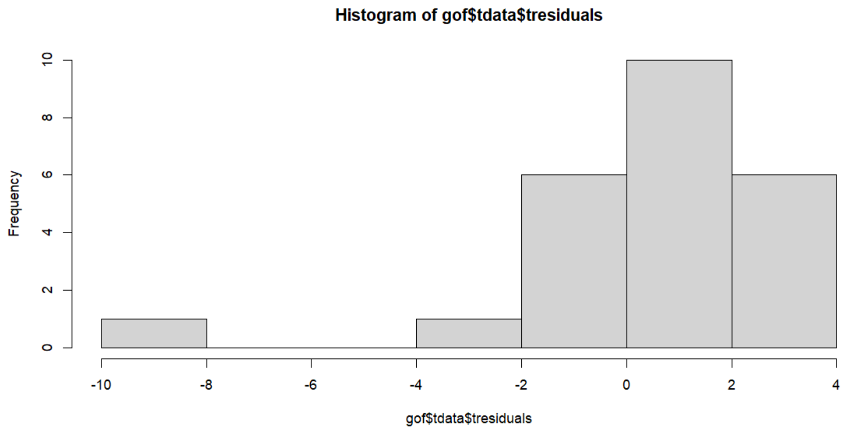

Figure 4: Histogram of residuals

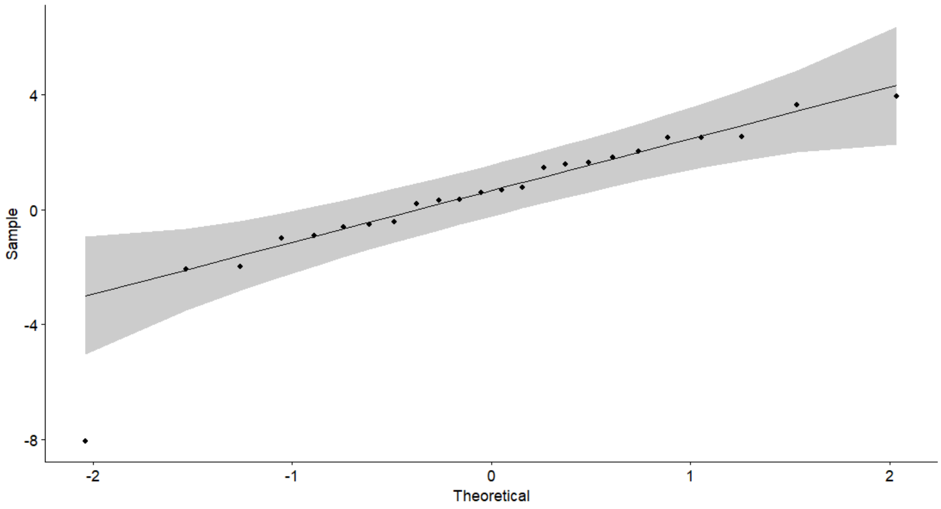

Figure 5: QQ plot of residuals

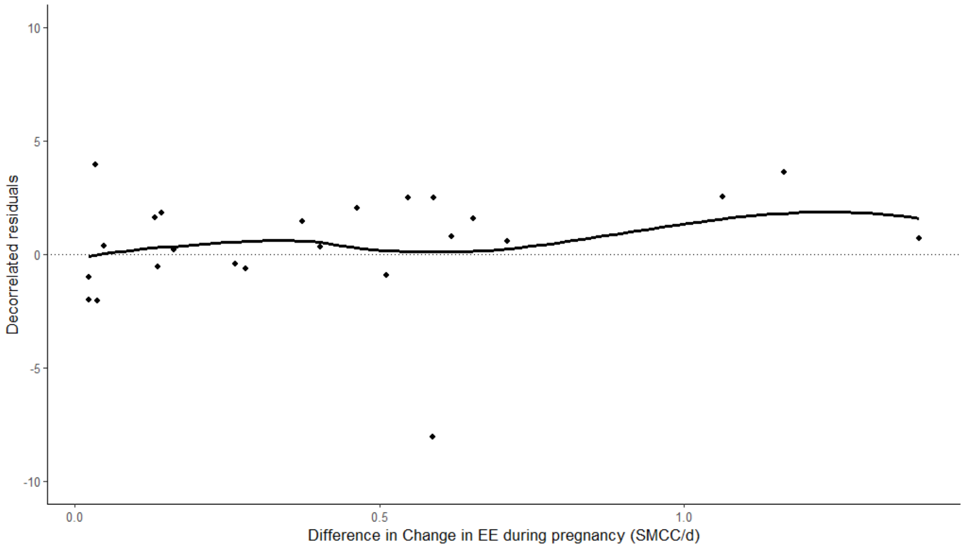

Figure 6: De-correlated residuals vs. exposure plot

Supplemental Material S10: Heterogeneity in the DRMA

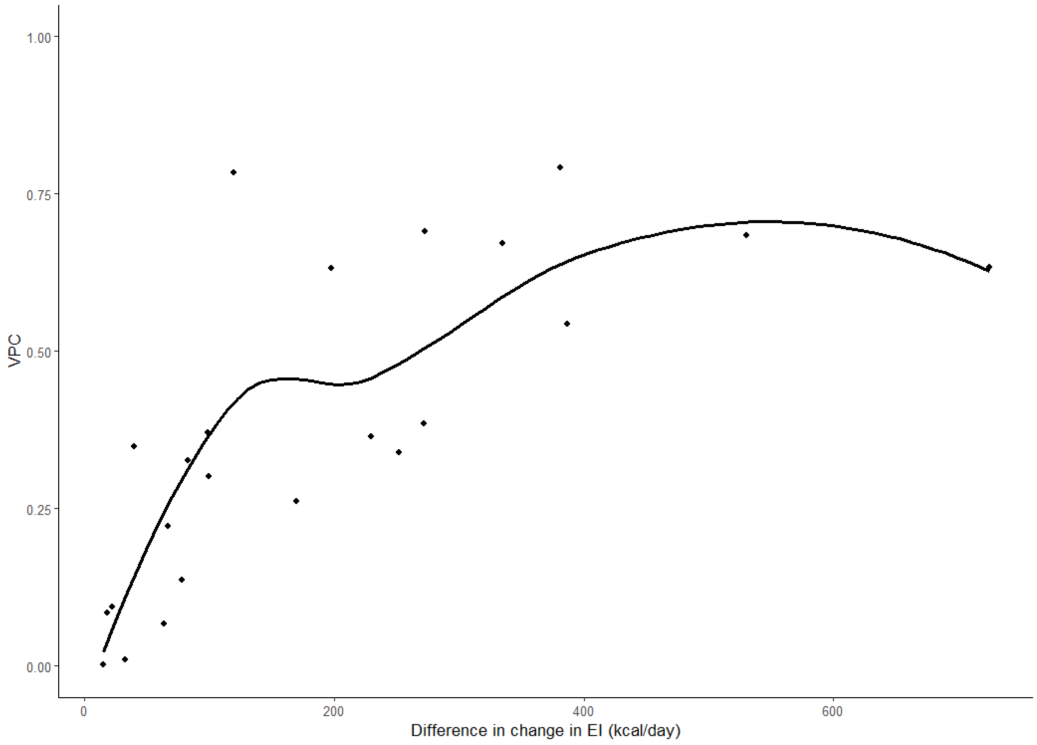

Figure 7: VPC plot for assessing between-study heterogeneity in DRMA of EI and GWG

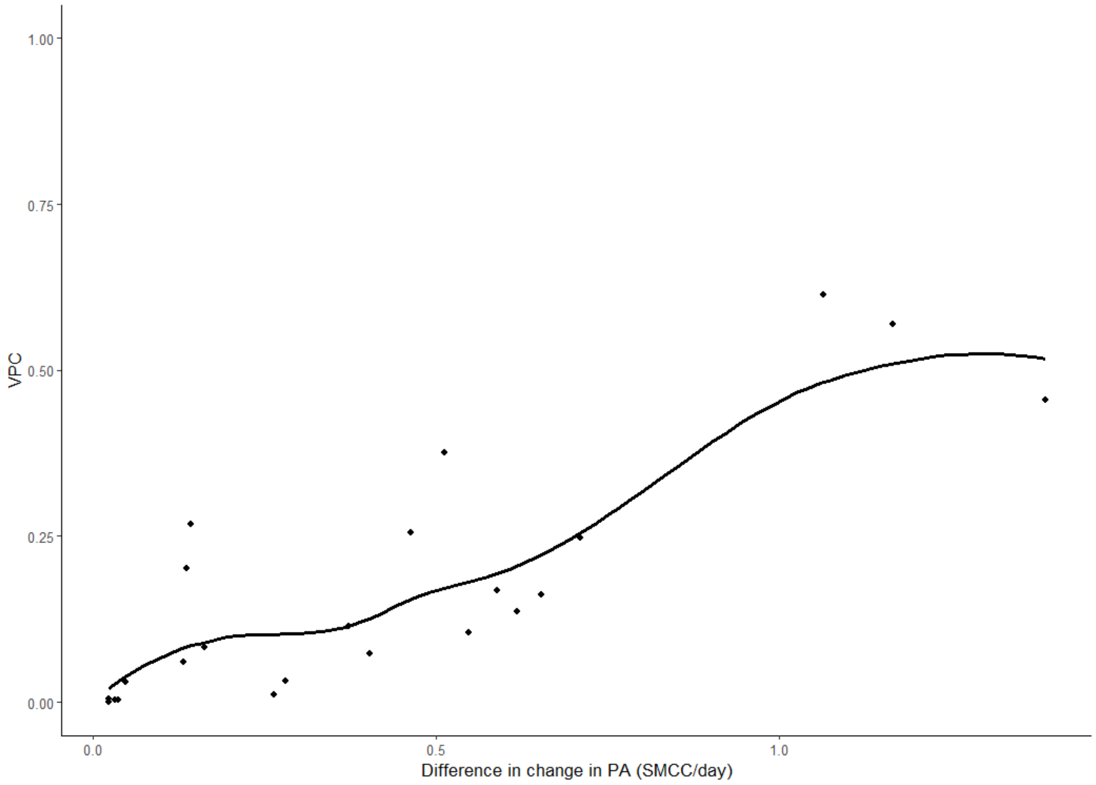

Figure 8: VPC plot for assessing between-study heterogeneity in DRMA of PA and GWG

Supplemental Material S11: DRMA and subgroup analyses of the association between changes in PA and GWG

Main analyses	Predicted change in GWG (kg)	95% Confidence Interval	P value for EI	AIC
**Per 100 kcal/day increase in EI**				
Crude	-0.24	-0.50 to 0.02	0.07	84.29
**Subgroup Analyses**	**Predicted change in GWG (kg)**	**95% Confidence Interval**	**P for subgroup difference**	**AIC**
Pre- / early pregnancy BMI				
All BMI [a]	-0.14	-0.41 to 0.12	0.15	84.52
Overweight / Obese	-0.50	-0.92 to -0.09		
Geographic location of study				
East	-0.51	-1.05 to 0.03	0.30	85.45
West [b]	-0.19	-0.44 to 0.06		
Risk of bias in measurement of PA				
High risk of bias	-0.23	-0.53 to 0.07	0.94	86.29
Low risk of bias	-0.25	-0.75 to 0.24		
Missing data				
< 10% missing	-0.79	-1.20 to -0.38	0.004	78.70
> 10% missing	-0.09	-0.31 to 0.13		
< 20% missing	-0.36	-0.70 to -0.13	0.32	83.51
> 20% missing	-0.10	-0.47 to 0.27		
GWG assessment method				
Self-reported pre-pregnancy weight	-0.28	-0.60 to 0.03	0.54	85.92
Measured early pregnancy weight	-0.10	-0.61 to 0.41		

[a] BMI was based on the inclusion/exclusion criteria of each study. One study included only women with normal BMI was grouped into the "All BMI" groups [b] A study conducted in Istanbul in Turkey was classified as "Western".[56] Abbreviations: AIC: Akaike Information Criterion, GWG: gestational weight gain, PA: physical activity, SMC: standardized mean change,

Supplemental Material S12: Leave-one-out analysis

Analyses of EI:

	StudyID	Coefficient	CI_lower	CI_upper	P_value
P	1	0.305	-0.029	0.640	0.073
P1	2	0.268	-0.097	0.633	0.150
P2	3	0.295	-0.021	0.610	0.067
P3	4	0.252	-0.086	0.590	0.143
P4	5	0.308	-0.030	0.645	0.074
P5	6	0.322	0.011	0.633	0.043
P6	7	0.286	-0.083	0.655	0.129
P7	8	0.256	-0.098	0.610	0.157
P8	9	0.333	0.028	0.638	0.032
P9	10	0.310	-0.044	0.664	0.086
P10	11	0.293	-0.016	0.601	0.063
P11	12	0.308	-0.049	0.664	0.091
P12	13	0.273	-0.053	0.598	0.101
P13	14	0.265	-0.035	0.565	0.083
P14	15	0.321	0.019	0.623	0.037
P15	17	0.388	0.204	0.572	0.000
P16	18	0.273	-0.064	0.609	0.112
P17	19	0.288	0.100	0.475	0.003
P18	20	0.289	-0.070	0.649	0.115
P19	21	0.292	-0.022	0.606	0.068

Analyses of PA:

	StudyID	Coefficient	CI_lower	CI_upper	P_value
P	1	-0.253	-0.532	0.026	0.075
P1	2	-0.195	-0.456	0.065	0.141
P2	3	-0.237	-0.499	0.025	0.076
P3	4	-0.230	-0.489	0.030	0.083
P4	5	-0.249	-0.521	0.022	0.072
P5	6	-0.228	-0.495	0.039	0.094
P6	7	-0.222	-0.535	0.091	0.165
P7	8	-0.190	-0.477	0.097	0.194
P8	9	-0.257	-0.516	0.003	0.053
P9	10	-0.286	-0.569	-0.003	0.048
P10	11	-0.278	-0.529	-0.027	0.030
P11	12	-0.262	-0.536	0.012	0.061
P12	13	-0.213	-0.494	0.067	0.136
P13	14	-0.234	-0.494	0.025	0.077
P14	15	-0.232	-0.492	0.028	0.081
P15	17	-0.166	-0.372	0.040	0.114
P16	18	-0.281	-0.534	-0.028	0.029
P17	19	-0.254	-0.510	0.002	0.052
P18	20	-0.233	-0.493	0.028	0.080
P19	21	-0.253	-0.519	0.012	0.061

Supplemental Material S13: Sensitivity analyses of DRMA of EI and PA with GWG

DRMA of EI and GWG	Accounting for cluster-RCTs:	Adjusting the sample sizes of cluster-RCT study groups using the ICC from the study reporting the largest ICC[49]:<br><br>0.30 kg additional GWG, 95% CI: -0.03 to 0.63.<br><br>Using the ICC from another study[50]:<br><br>0.30 kg additional GWG, 95% CI: -0.02 to 0.62.<br><br>Excluding cluster-RCTs<br><br>0.29 kg additional GWG, 95% CI: -0.09 to 0.67.
	Timing of EI and GWG assessment	Excluding studies that assessed EI only to mid-pregnancy:[51,52]<br><br>0.31 kg additional total GWG, 95% CI: -0.03 to 0.65 kg.<br><br>Excluding studies that assessed GWG only to mid-pregnancy:[51]<br><br>0.33 kg additional total GWG, 95% CI: 0.03 to 0.64 kg.
DRMA PA and GWG	Different correlation coefficients for estimating variance of SMCC:	1 (original): -0.24 kg, 95% CI: -0.50 to 0.02, p= 0.072<br><br>2: -0.13 kg, 95% CI: -0.39 to 0.13, p=0.341<br><br>3: -0.18 kg, 95% CI: -0.37 to 0.01, p=0.063<br><br>4: -0.36 kg, 95% CI: -0.73 to 0.02, p=0.061
	Accounting for cluster-RCTs:	Adjusting the sample sizes of cluster-RCT study groups using the ICC from the study reporting the largest ICC[49]:<br><br>0.23 kg less GWG, 95% CI: -0.51 to 0.05.<br><br>Using the ICC from another study,[50]<br><br>0.24 kg less GWG, 95% CI: -0.51 to 0.03.<br><br>Excluding cluster-RCTs<br><br>0.23 kg less GWG, 95% CI: -0.52 to 0.06.
	Timing of assessment of PA and GWG	Excluding studies that assessed PA only to mid-

		pregnancy[51,52]:<br><br>0.30 kg less total GWG, 95% CI: -0.55 to -0.05 kg.<br><br>Excluding studies that assessed GWG only to mid-pregnancy[51]:<br><br>0.26 kg less total GWG, 95% CI: -0.52 to 0.00 kg.

Supplemental Material S14: Strengths and limitations of using SMC for PA:

One other key strength of our study is that we employed SMC as the estimate of change in PA over the course of pregnancy, so we were able to synthesize data from all studies, regardless of the metric used (e.g., steps per day, physical activity score, etc.), thereby increasing the number and representativeness of included studies.

However, the employment of SMC for PA is not without limitations. Theoretically, the use of the SMC is justified, as measures of change across studies of the same construct using different scales may be interpreted as linear transformations of each other.[57] However, to use the SD as a scaling factor, between-study variation in SD is assumed to only reflect differences in measurement scales, and not differences in the reliability of measurement tools, or true variability among study designs[57-59]. If the variance in PA at baseline or follow-up differs between primary studies, studies that enrolled a more heterogeneous group of women will yield smaller SMCs than those enrolling a more homogeneous population, even if the raw mean difference was identical.[60]

Regarding alternative estimates of changes in EI and PA we could have used, while some studies reported usable estimates such as changes from baseline, directly adjusted for baseline values, irreconcilable differences between studies prevented us from using these values in our analyses.

References

1. Abeysekera MV, Morris JA, Davis GK, O'Sullivan AJ. Alterations in energy homeostasis to favour adipose tissue gain: A longitudinal study in healthy pregnant women. *Aust N Z J Obstet Gynaecol*. Feb 2016;56(1):42-8. doi:10.1111/ajo.12398

2. Berggren EK, O'Tierney-Ginn P, Lewis S, Presley L, De-Mouzon SH, Catalano PM. Variations in resting energy expenditure: impact on gestational weight gain. *Am J Obstet Gynecol*. Oct 2017;217(4):445 e1-445 e6. doi:10.1016/j.ajog.2017.05.054

3. Bergmann MM, Flagg EW, Miracle-McMahill HL, Boeing H. Energy intake and net weight gain in pregnant women according to body mass index (BMI) status. *Int J Obes Relat Metab Disord*. Nov 1997;21(11):1010-7. doi:10.1038/sj.ijo.0800509

4. Blumfield ML, Schreurs M, Rollo ME, MacDonald-Wicks LK, Kokavec A, Collins CE. The association between portion size, nutrient intake and gestational weight gain: a secondary analysis in the WATCH study 2006/7. *J Hum Nutr Diet*. Jun 2016;29(3):271-80. doi:10.1111/jhn.12330

5. Catalano PM, Roman-Drago NM, Amini SB, Sims EA. Longitudinal changes in body composition and energy balance in lean women with normal and abnormal glucose tolerance during pregnancy. *Am J Obstet Gynecol*. Jul 1998;179(1):156-65. doi:10.1016/s0002-9378(98)70267-4

6. Deierlein AL, Siega-Riz AM, Herring A. Dietary energy density but not glycemic load is associated with gestational weight gain. *Am J Clin Nutr*. Sep 2008;88(3):693-9. doi:10.1093/ajcn/88.3.693

7. Diemert A, Lezius S, Pagenkemper M, et al. Maternal nutrition, inadequate gestational weight gain and birth weight: results from a prospective birth cohort. *BMC Pregnancy Childbirth*. Aug 15 2016;16:224. doi:10.1186/s12884-016-1012-y

8. Dodd JM, Cramp C, Sui Z, et al. The effects of antenatal dietary and lifestyle advice for women who are overweight or obese on maternal diet and physical activity: the LIMIT randomised trial. *BMC Med*. Oct 13 2014;12:161. doi:10.1186/s12916-014-0161-y

9. Gaillard R, Durmus B, Hofman A, Mackenbach JP, Steegers EA, Jaddoe VW. Risk factors and outcomes of maternal obesity and excessive weight gain during pregnancy. *Obesity (Silver Spring)*. May 2013;21(5):1046-55. doi:10.1002/oby.20088

10. Gilmore LA, Butte NF, Ravussin E, Han H, Burton JH, Redman LM. Energy Intake and Energy Expenditure for Determining Excess Weight Gain in Pregnant Women. *Obstet Gynecol*. May 2016;127(5):884-892. doi:10.1097/AOG.0000000000001372

11. Gunther J, Hoffmann J, Kunath J, et al. Effects of a Lifestyle Intervention in Routine Care on Prenatal Dietary Behavior-Findings from the Cluster-Randomized GeliS Trial. *J Clin Med*. Jul 2 2019;8(7)doi:10.3390/jcm8070960

12. Jebeile H, Mijatovic J, Louie JCY, Prvan T, Brand-Miller JC. A systematic review and metaanalysis of energy intake and weight gain in pregnancy. *American journal of obstetrics and gynecology*. 2016;214(4):465-483.

13. Karamanos B, Thanopoulou A, Anastasiou E, et al. Relation of the Mediterranean diet with the incidence of gestational diabetes. *Eur J Clin Nutr*. Jan 2014;68(1):8-13. doi:10.1038/ejcn.2013.177

14. Lagiou P, Tamimi RM, Mucci LA, Adami HO, Hsieh CC, Trichopoulos D. Diet during pregnancy in relation to maternal weight gain and birth size. *Eur J Clin Nutr*. Feb 2004;58(2):231-7. doi:10.1038/sj.ejcn.1601771

15. Lai JS, Soh SE, Loy SL, et al. Macronutrient composition and food groups associated with gestational weight gain: the GUSTO study. *Eur J Nutr*. Apr 2019;58(3):1081-1094. doi:10.1007/s00394-018-1623-3

16. Lawrence M, Lawrence F, Coward WA, Cole TJ, Whitehead RG. Energy requirements of pregnancy in The Gambia. *Lancet*. Nov 7 1987;2(8567):1072-6. doi:10.1016/s0140-6736(87)91492-9

17. Most J, Amant MS, Hsia DS, et al. Evidence-based recommendations for energy intake in pregnant women with obesity. *J Clin Invest*. Aug 1 2019;129(11):4682-4690. doi:10.1172/JCI130341

18. Najpaverova S, Kovarik M, Kacerovsky M, Zadak Z, Hronek M. The Relationship of Nutritional Energy and Macronutrient Intake with Pregnancy Outcomes in Czech Pregnant Women. *Nutrients*. Apr 20 2020;12(4)doi:10.3390/nu12041152

19. Phelan S, Wing RR, Brannen A, et al. Randomized controlled clinical trial of behavioral lifestyle intervention with partial meal replacement to reduce excessive gestational weight gain. *Am J Clin Nutr*. Feb 1 2018;107(2):183-194. doi:10.1093/ajcn/nqx043

20. Piers LS, Diggavi SN, Thangam S, van Raaij JM, Shetty PS, Hautvast JG. Changes in energy expenditure, anthropometry, and energy intake during the course of pregnancy and lactation in well-nourished Indian women. *Am J Clin Nutr*. Mar 1995;61(3):501-13. doi:10.1093/ajcn/61.3.501

21. Poston L, Bell R, Croker H, et al. Effect of a behavioural intervention in obese pregnant women (the UPBEAT study): a multicentre, randomised controlled trial. *Lancet Diabetes Endocrinol*. Oct 2015;3(10):767-77. doi:10.1016/S2213-8587(15)00227-2

22. Rodrigues PL, Lacerda EM, Schlussel MM, Spyrides MH, Kac G. Determinants of weight gain in pregnant women attending a public prenatal care facility in Rio de Janeiro, Brazil: a prospective study, 2005-2007. *Cad Saude Publica*. 2008;24 Suppl 2:S272-84. doi:10.1590/s0102-311x2008001400012

23. Rugina C, Marginean CO, Melit LE, Giga DV, Modi V, Marginean C. Relationships between excessive gestational weight gain and energy and macronutrient intake in pregnant women. *J Int Med Res*. Aug 2020;48(8):300060520933808. doi:10.1177/0300060520933808

24. Savard C, Lebrun A, O'Connor S, Fontaine-Bisson B, Haman F, Morisset AS. Energy expenditure during pregnancy: a systematic review. *Nutr Rev*. Mar 9 2021;79(4):394-409. doi:10.1093/nutrit/nuaa093

25. Siega-Riz AM, Adair LS. Biological determinants of pregnancy weight gain in a Filipino population. *Am J Clin Nutr*. Mar 1993;57(3):365-72. doi:10.1093/ajcn/57.3.365

26. Streuling I, Beyerlein A, Rosenfeld E, Schukat B, von Kries R. Weight gain and dietary intake during pregnancy in industrialized countries--a systematic review of observational studies. *J Perinat Med*. Mar 2011;39(2):123-9. doi:10.1515/jpm.2010.127

27. Stuebe AM, Oken E, Gillman MW. Associations of diet and physical activity during pregnancy with risk for excessive gestational weight gain. *Am J Obstet Gynecol*. Jul 2009;201(1):58 e1-8. doi:10.1016/j.ajog.2009.02.025

28. Thongprasert K, Tanphaichitre V, Valyasevi A, Kittigool J, Durnin JV. Energy requirements of pregnancy in rural Thailand. *Lancet*. Oct 31 1987;2(8566):1010-2. doi:10.1016/s0140-6736(87)92568-2

29. Tielemans MJ, Garcia AH, Peralta Santos A, et al. Macronutrient composition and gestational weight gain: a systematic review. *Am J Clin Nutr*. Jan 2016;103(1):83-99. doi:10.3945/ajcn.115.110742

30. Uusitalo U, Arkkola T, Ovaskainen ML, et al. Unhealthy dietary patterns are associated with weight gain during pregnancy among Finnish women. *Public Health Nutr*. Dec 2009;12(12):2392-9. doi:10.1017/S136898000900528X

31. Van Horn L, Peaceman A, Kwasny M, et al. Dietary Approaches to Stop Hypertension Diet and Activity to Limit Gestational Weight: Maternal Offspring Metabolics Family

Intervention Trial, a Technology Enhanced Randomized Trial. *Am J Prev Med*. Nov 2018;55(5):603-614. doi:10.1016/j.amepre.2018.06.015

32. van Raaij JM, Vermaat-Miedema SH, Schonk CM, Peek ME, Hautvast JG. Energy requirements of pregnancy in The Netherlands. *Lancet*. Oct 24 1987;2(8565):953-5. doi:10.1016/s0140-6736(87)91431-0

33. Yang L, Wu C, Bao Y, et al. [Energy intake as determinants of gestational weight gain in Chengdu]. *Wei Sheng Yan Jiu*. Nov 2018;47(6):895-905.

34. Higgins JPT, Thomas J, Chandler J, et al. Chapter 6: Choosing effect measures and computing estimates of effect. In: Higgins JPT, Li T, JJ D, eds. *Cochrane Handbook for Systematic Reviews of Interventions version 63*. Cochrane; 2022.

35. Cai S, Zhou J, Pan J. Estimating the sample mean and standard deviation from order statistics and sample size in meta-analysis. *Stat Methods Med Res*. Dec 2021;30(12):2701-2719. doi:10.1177/09622802211047348

36. Liu J, Wilcox S, Wingard E, Turner-McGrievy G, Hutto B, Burgis J. A Behavioral Lifestyle Intervention to Limit Gestational Weight Gain in Pregnant Women with Overweight and Obesity. *Obesity (Silver Spring)*. Apr 2021;29(4):672-680. doi:10.1002/oby.23119

37. Ferrara A, Hedderson MM, Brown SD, et al. A telehealth lifestyle intervention to reduce excess gestational weight gain in pregnant women with overweight or obesity (GLOW): a randomised, parallel-group, controlled trial. *Lancet Diabetes Endocrinol*. Jun 2020;8(6):490-500. doi:10.1016/S2213-8587(20)30107-8

38. Crippa A, Discacciati A, Bottai M, Spiegelman D, Orsini N. One-stage dose–response meta-analysis for aggregated data. *Statistical Methods in Medical Research*. 2019;28(5):1579-1596. doi:10.1177/0962280218773122

39. Discacciati A, Crippa A, Orsini N. Goodness of fit tools for dose-response meta-analysis of binary outcomes. *Res Synth Methods*. Jun 2017;8(2):149-160. doi:10.1002/jrsm.1194

40. Vetter TR. Fundamentals of Research Data and Variables: The Devil Is in the Details. *Anesth Analg*. Oct 2017;125(4):1375-1380. doi:10.1213/ane.0000000000002370

41. Orsini N, Bellocco R, Greenland S. Generalized Least Squares for Trend Estimation of Summarized Dose–response Data. *The Stata Journal*. 2006;6(1):40-57. doi:10.1177/1536867x0600600103

42. Greenland S, Longnecker MP. Methods for Trend Estimation from Summarized Dose-Response Data, with Applications to Meta-Analysis. *American Journal of Epidemiology*. 1992;135(11):1301-1309. doi:10.1093/oxfordjournals.aje.a116237

43. Berlin JA, Longnecker MP, Greenland S. Meta-analysis of epidemiologic dose-response data. *Epidemiology*. May 1993;4(3):218-28. doi:10.1097/00001648-199305000-00005

44. Crippa A, Orsini N. Dose-response meta-analysis of differences in means. *BMC Medical Research Methodology*. 2016/08/02 2016;16(1):91. doi:10.1186/s12874-016-0189-0

45. Shim SR, Lee J. Dose-response meta-analysis: application and practice using the R software. *Epidemiol Health*. 2019;41:e2019006. doi:10.4178/epih.e2019006

46. Vinceti M, Filippini T, Malavolti M, et al. Dose-response relationships in health risk assessment of nutritional and toxicological factors in foods: development and application of novel biostatistical methods. *EFSA Supporting Publications*. 2020;17(7):1899E. doi:https://doi.org/10.2903/sp.efsa.2020.EN-1899

47. Müller S, Scealy JL, Welsh AH. Model Selection in Linear Mixed Models. *Statistical Science*. 2013;28(2):135-167, 33.

48. Higgins JPT ES, Li T. Chapter 23: Including variants on randomized trials. In: Higgins JPT TJ, Chandler J, Cumpston M, Li T, Page MJ, Welch VA ed. *Cochrane Handbook for Systematic Reviews of Interventions version 63*. Cochrane; 2022. https://training.cochrane.org/handbook/current/chapter-23

49. Luoto RM, Kinnunen TI, Aittasalo M, et al. Prevention of Gestational Diabetes: Design of a Cluster-Randomized Controlled Trial and One-Year Follow-Up. *BMC Pregnancy and Childbirth*. 2010/08/03 2010;10(1):39. doi:10.1186/1471-2393-10-39

50. Kunath J, Günther J, Rauh K, et al. Effects of a lifestyle intervention during pregnancy to prevent excessive gestational weight gain in routine care – the cluster-randomised GeliS trial. *BMC Medicine*. 2019/01/14 2019;17(1):5. doi:10.1186/s12916-018-1235-z

51. Jing W, Huang Y, Liu X, Luo B, Yang Y, Liao S. The effect of a personalized intervention on weight gain and physical activity among pregnant women in China. *Int J Gynaecol Obstet*. May 2015;129(2):138-41. doi:10.1016/j.ijgo.2014.11.014

52. Huang RC, Silva D, Beilin L, et al. Feasibility of conducting an early pregnancy diet and lifestyle e-health intervention: the Pregnancy Lifestyle Activity Nutrition (PLAN) project. *J Dev Orig Health Dis*. Feb 2020;11(1):58-70. doi:10.1017/S2040174419000400

53. Hui A, Back L, Ludwig S, et al. Lifestyle intervention on diet and exercise reduced excessive gestational weight gain in pregnant women under a randomised controlled trial. *BJOG*. Jan 2012;119(1):70-7. doi:10.1111/j.1471-0528.2011.03184.x

54. Hui AL, Back L, Ludwig S, et al. Effects of lifestyle intervention on dietary intake, physical activity level, and gestational weight gain in pregnant women with different pre-pregnancy Body Mass Index in a randomized control trial. *BMC Pregnancy Childbirth*. Sep 24 2014;14:331. doi:10.1186/1471-2393-14-331

55. Hajian S, Aslani A, Sarbakhsh P, Fathnezhad-Kazemi A. The effectiveness of healthy lifestyle interventions on weight gain in overweight pregnant women: A cluster-randomized controlled trial. Article. *Nurs Open*. Nov 2020;7(6):1876-1886. doi:10.1002/nop2.577

56. Aşcı Ö, Rathfisch G. Effect of lifestyle interventions of pregnant women on their dietary habits, lifestyle behaviors, and weight gain: a randomized controlled trial. *Journal of Health, Population and Nutrition*. 2016;35:1-9.

57. Murad MH, Wang Z, Chu H, Lin L. When continuous outcomes are measured using different scales: guide for meta-analysis and interpretation. *BMJ*. Jan 22 2019;364:k4817. doi:10.1136/bmj.k4817

58. Greenland S, Maclure M, Schlesselman JJ, Poole C, Morgenstern H. Standardized Regression Coefficients: A Further Critique and Review of Some Alternatives. *Epidemiology*. 1991;2(5):387-392.

59. Greenland S, Schlesselman JJ, Criqui MH. The fallacy of employing standardized regression coefficients and correlations as measures of effect. *Am J Epidemiol*. Feb 1986;123(2):203-8. doi:10.1093/oxfordjournals.aje.a114229

60. Thorlund K, Walter SD, Johnston BC, Furukawa TA, Guyatt GH. Pooling health-related quality of life outcomes in meta-analysis-a tutorial and review of methods for enhancing interpretability. *Res Synth Methods*. Sep 2011;2(3):188-203. doi:10.1002/jrsm.46
